# Diverse Polyphenols from *Hypericum faberi*

**DOI:** 10.1007/s13659-019-0206-1

**Published:** 2019-05-09

**Authors:** Xin-Wen Zhang, Yan-Song Ye, Fan Xia, Xing-Wei Yang, Gang Xu

**Affiliations:** 1 State Key Laboratory of Phytochemistry and Plant Resources in West China, Kunming Institute of Botany, Chinese Academy of Sciences, Yunnan Key Laboratory of Natural Medicinal Chemistry, Kunming, 650201 People’s Republic of China; 2 University of Chinese Academy of Sciences, Beijing, 100049 People’s Republic of China

**Keywords:** *Hypericum faberi*, Isoprenylated xanthone, Isoprenylated acylphloroglucinol, Chromone, Cytotoxicitiy

## Abstract

**Abstract:**

Six new polyphenols with different isoprenylated xanthones, isoprenylated acylphloroglucinols, and chromone architectures, hyperfaberols A–F (**1**–**6**), were isolated from the whole plants of *Hypericum faberi* along with seven other related known compounds. In which hyperfaberols A/B (**1**/**2**) and **12**–**13** were isoprenylated xanthones, hyperfaberols C–E (**3**–**5**) and **8**–**11** were seven isoprenylated acylphloroglucinol derivatives, while **6**–**7** were two chromones. Their structures were elucidated by comprehensive analysis of their spectroscopic data as well as detailed comparison with the literature data. Compounds **1** and **11** showed cytotoxities against the human esophageal cancer cell line (ECA-109) and the pancreatic tumor cell line (PANC-1) in vitro, respectively.

**Graphical Abstract:**

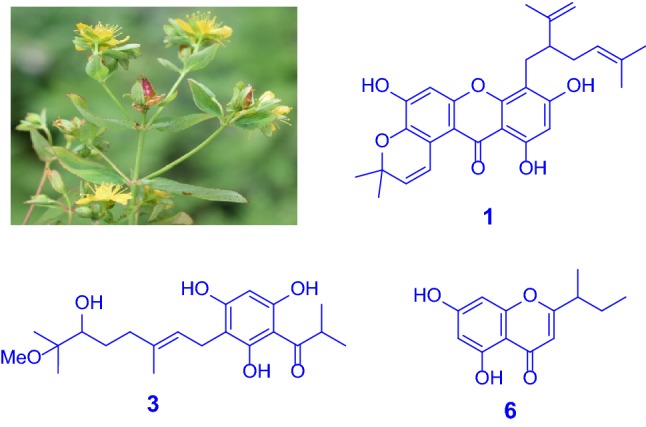

**Electronic supplementary material:**

The online version of this article (10.1007/s13659-019-0206-1) contains supplementary material, which is available to authorized users.

## Introduction

The genus *Hypericum* (Hypericaceae Juss.) comprises nearly 500 species and is subdivided into 36 taxonomic sections [1]. The plants of this genus have been proved to be rich sources of prenylated phenolic metabolites with diverse phloroglucinols, xanthones, and flavonoids structures as well as valuable bioactivities such as antidepressant, antibacterial, and antiproliferative activities [2–4]. Our group has been engaged in the systematic studies of isoprenylated polyphenols from *Hypericum* species for more than ten years, and has discovered a series of fascinating chemical structures with intriguing biological activities [5–15]. *Hypericum faberi* R. Keller is distributed in the south and west of China [16]. Detailed literature research shows that it has never been phytochemical studied before. To further explore the new and bioactive natural polyphenols from *Hypericum* species, *H. faberi* was selected to carry out a phytochemical study and six new polyphenols, hyperfaberols A–F (**1**–**6**), together with additional seven known related derivatives 5,7-dihydroxy-2-isopropylchromon (**7**) [17], madeleinol A (**8**) [18], empetrifranzinans A/B (**9**/**10**) [18], otogirin (**11**) [19], dulxanthone D (**12**) [20], and toxyloxanthone B (**13**) [21] were isolated. It’s noteworthy that these isolates can be divided to three group based on their structural characters: isoprenylated xanthones (**1**–**2** and **12**–**13**), isoprenylated acylphloroglucinols (**3**–**5** and **8**–**11**), and chromones (**6**–**7**). In the bioactive assay, compounds **1** and **11** showed cytotoxities against the human esophageal cancer cell line (ECA-109) and the pancreatic tumor cell line (PANC-1) in vitro, respectively. Herein, we report the results of the isolates from the whole plant of *H. faberi* and their biological evaluation.

## Results and Discussion

Hyperfaberol A (**1**) was obtained as a yellow amorphous powder. The HRESIMS data showed a deprotonated molecular ion at *m/z* 461.1979 [M–H]^−^, indicating a molecular formula of C_28_H_30_O_6_ (calcd for C_28_H_29_O_6_, 461.1970). The IR spectrum showed absorption bands of hydroxy (3423 cm^−1^) and conjugated carbonyl (1634 cm^−1^) functionalities. The ^1^H NMR spectrum showed a hydroxy group (*δ*_H_ 13.24), two aromatic protons (*δ*_H_ 6.14 and 6.73), two protons of a terminal double bond (*δ*_H_ 4.37, 4.48, 1H each, s), and five methyls (*δ*_H_ 1.34–1.64, s) (Table 1). The ^13^C and DEPT NMR data showed 28 carbon signals which were sorted by HSQC techniques as five methyls, three methylenes (including one terminal double bond, *δ*_C_ 111.0 and 147.2), six methines, and 14 quaternary carbons. The typical thirteen signals for a carbonyl (*δ*_C_ 181.6) and two *penta*-substitued phenyl units (*δ*_C_ 162.5, 160.3, 154.0, 153.5, 152.4, 138.2, 119.5, 106.0, 104.5, 102.4, 102.0, 97.1) can be easiliy distunguished in the ^13^C NMR, which indicated a xanthone skeleton of **1** as hinted by the UV absorption bands at 203, 267 and 333 [22, 23]. In the HMBC spectrum, the correlations from 1-HO to C-1, C-2, C-9, and C-9a; from H-2 to C-1, C-3, C-4, C-9, and C-9a; from H-5 to C-6, C-7, C-8a, C-9, and C-10a, and from H_2_-11 to C-3, C-4, and C-4a confirmed the xanthone framework (Fig. 2).

**Table 1:** ^13^C (150 MHz) and ^1^H (600 MHz) NMR Data of **1** (in DMSO)

No.	*δ*_C_ (type)	*δ*_H_ (*J* in Hz)	No.	*δ*_C_ (type)	*δ*_H_ (*J* in Hz)
**1**	160.3 (C)		13	31.0 (CH_2_)	2.02, m
**2**	97.1 (CH)	6.14, s	14	123.0 (CH)	4.96, t (6.9)
**3**	162.5 (C)		15	130.9 (C)	
**4**	104.5 (C)		16	25.5 (CH_3_)	1.55, s
**4a**	154.0 (C)		17	17.6 (CH_3_)	1.45, s
**10a**	153.5 (C)		18	147.2 (C)	
**5**	102.4 (CH)	6.73, s	19	18.4 (CH_3_)	1.64, s
**6**	152.4 (C)		20	111.0 (CH_2_)	4.37, s
**7**	138.2 (C)				4.48, s
**8**	119.5 (C)		21	120.3 (CH)	7.82, d (10.2)
**8a**	106.0 (C)		22	132.6 (CH)	5.86, d (10.2)
**9**	181.6 (C)		23	75.0 (C)	
**9a**	102.0 (C)		24	26.6 (CH_3_)	1.34, s
**11**	26.4 (CH_2_)	2.65, m	25	26.6 (CH_3_)	1.34, s
**12**	46.9 (CH)	2.35, m	HO-1		13.24, s

**Fig. 1:**
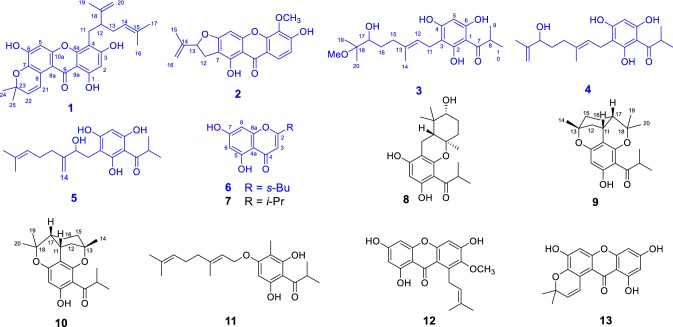
Structures of **1**–**13**

Carefully analysis of the 1D NMR spectra indicated the remianing 15 carbon signals (from C-11 to C-25) should assigned to three isoprenyl derived units. Two of which were deduced to form a lavandulyl moiety by the ^1^H-^1^H COSY cross-peaks of H_2_-11/H-12/H_2_-13/H-14 together with the HMBC correlations from H_2_-20 to C-12, C-18 and C-19, and from H_3_-16/H_3_-17 to C-14 and C-15. The location of this lavandulyl moiety at C-4 was evidenced by the HMBC correlations of H-11 with C-3, C-4, and C-4a [24]. The third isoprenyl group was elucidated to form a characteristic pyran ring between C-7 and C-8 as revealed by the HMBC correlations from H-21 to C-7, C-8, C-8a, and C-23, and from H_3_-24/H_3_-25 to C-7, C-22, and C-23. Consequently, the structure of **1**, hypefaberol A, was established as shown (Fig. 1).

Hyperfaberol B (**2**), a yellow amorphous powder, had the molecular formula C_19_H_16_O_6_ as deduced from its HRESIMS *m/z* 339.0881 [M–H]^−^ (calcd for 339.0874) and ^13^C NMR data. The IR spectrum showed absorption bands of hydroxy (3370 cm^−1^) and conjugated carbonyl (1664 cm^−1^). The ^1^H NMR revealed the presence of one hydroxy (*δ*_H_ 13.15, s), three aromatic protons [*δ*_H_ 6.43 (1H, s), 6.97 (1H, d, *J* = 8.8 Hz), and 7.90 (1H, d, *J* = 8.8 Hz)], two protons of a terminal double bond (*δ*_H_ 4.94, 5.09, 1H each, s), one methyl (*δ*_H_ 1.76, 3H, s), and one methoxy group (*δ*_H_ 4.09, 3H, s) (Table 2). The ^13^C NMR spectrum exhibited 19 signals, including 15 sp^2^ carbons [one carbonyl, 12 aromatic carbons, and a terminal double bond signals (*δ*_C_ 113.0 and 143.3)] and four sp^3^ carbons (one methyl, one methoxy, one methylene, and one oxygenated methine). Side-by-side analysis of the ^1^H and ^13^C NMR spectra indicated that **2** were closely similar to the known xanthone, 2,3-dihydro-4-hydroxy-9-methoxy-2-(1-methylethenyl)-6*H*-furo[3,2-b]xanthen-5-one [25]. The only difference was that the aromatic proton (H-3) in the latter was replaced by a hydroxy group in **2**, which can be confirmed by the downfiled chemical shift of 154.3 (C-3), the obvious HMBC correlations from the two *ortho*-coupled aromatic protons H-1/H-2 to C-3, from H-1 to C-9, C-9a, and C-4a, from H-2 to C-3, C-4, C-9, and C-9a, from H-5 to C-6, C-7, C-8a, C-9, and C-10a, from HO-8 to C-7, C-8, C-8a, and C-9, from H_2_-12 to C-6, C-7, and C-8, from H_2_-16 to C-13, C-14, and C-15, and the ^1^H-^1^H COSY between H_2_-12 and H-13. Thus, the structure of **2** was elucidated and named hyperfaberol B (Fig. 1).

**Table 2:** ^13^C (150 MHz) and ^1^H (600 MHz) NMR Data of **2** (in CDCl_3_)

No.	*δ*_C_ (type)	*δ*_H_ (*J* in Hz)	No.	*δ*_C_ (type)	*δ*_H_ (*J* in Hz)
**1**	122.0 (CH)	7.90, d (8.8)	9	180.4 (C)	
**2**	112.5 (CH)	6.97, d (8.8)	9a	115.1 (C)	
**3**	154.3 (C)		11	62.2 (CH_3_)	4.09, s
**4**	133.8 (C)		12	30.7 (CH_2_)	3.02, dd (15.4, 7.4)
**4a**	149.7 (C)				3.37, dd (15.4, 9.7)
**10a**	157.9 (C)		13	88.6 (CH)	5.34, dd (9.4, 7.7)
**5**	89.5 (CH)	6.43, s	14	143.3 (C)	
**6**	167.3 (C)		15	17.2 (CH_3_)	1.76, s
**7**	108.0 (C)		16	113.0 (CH_2_)	4.94, s
**8**	158.2 (C)				5.09, s
**8a**	103.6 (C)		HO-8		13.15, s

**Fig. 2:**
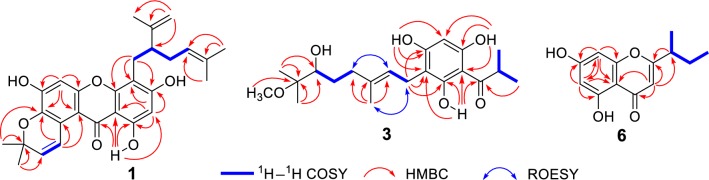
Key HMBC, ^1^H–^1^H COSY, and ROESY correlations of **1**, **3**, and **6**

Hyperfaberol C (**3**) was isolated as a yellow gum. The molecular formula was established as C_21_H_32_O_6_ from its ^13^C NMR (Table 3) and HRESIMS data (*m/z* 379.2136, [M–H]^−^, calcd for 379.2126). The IR absorptions implied hydroxy (3417 cm^−1^) and carbonyl (1622 cm^−1^) functionalities. Its ^1^H NMR spectrum showed three hydroxy groups (*δ*_H_ 10.26, 10.51 and 14.14), an isopropyl group (*δ*_H_ 1.05, 6H, d, *J* = 6.7 Hz; 3.89, sept., *J* = 6.7 Hz), three singlet methyls (*δ*_H_ 0.94, 1.01, and 1.66), a methoxy group (*δ*_H_ 3.03), one olefinic proton (*δ*_H_ 5.11), and one aromatic proton (*δ*_H_ 5.97). The ^13^C and DEPT NMR spectrum showed 21 carbon signals including 10 signals assignable to an isobutyryl (*δ*_C_ 209.5, C-7; 38.0, C-8; and 19.3, C-9/10) and a phloroglucinol core (*δ*_C_ 102.7, C-1; 163.8, C-2; 106.0, C-3; 162.2, C-4; 94.2, C-5; and 159.6, C-6), which indicated that **3** could be an acylphloroglucinol derivative. The location of the acyl group at C-1 was evidenced by the HMBC correlation of HO-2 with C-7 and further indicated the formation of intramolecular hydrogen bonding between the hydroxyl and the carbonyl group. In addition, the HMBC correlations from HO-2 to C-1, C-2, and C-3, from HO-4 to C-3, C-4, and C-5, and from HO-6 to C-1, C-5, and C-6 were all presented, which confirmed the phloroglucinol core furthermore. The remaining eleven signals was assigned to three methyls, one methoxy, three methylenes, two methines (including one sp^2^ methine), and two quaternary carbons (including one sp^2^ carbon). The ^1^H-^1^H COSY correlations of H_2_-11/H-12 and H_2_-15/H_2_-16/H-17, conjugated with the HMBC correlations from H_3_-14 to C-12, C-13, and C-15, from H_3_-19/H_3_-20 to C-17 and C-18, and from H_3_-21 to C-18, established the 6-hydroxy-7-methoxy-3,7-dimethyl-2-octenyl partial structure for the fifteen signals moiety (Fig. 2) [26]. Moreover, the HMBC correlations from H_2_-11 to C-2, C-3 and C-4 established the attachment of the moiety at C-3. The NOE correlations of H_2_-11/Me-14 and H_2_-12/H_2_-15 in the ROESY spectrum elucidated the 12*E*-configured double bond of the moiety. Thus, the structure of hyperfaberol C (**3**) was elucidated (Fig. 1).

**Table 3:** ^13^C (150 MHz) and ^1^H (600 MHz) NMR Data of **3** and **4** (in DMSO)

No.	**3**		**4**	
	*δ*_C_ (type)	*δ*_H_ (*J* in Hz)	*δ*_C_ (type)	*δ*_H_ (*J* in Hz)
1	102.7 (C)		102.6 (C)	
2	163.8 (C)		163.8 (C)	
3	106.0 (C)		105.9 (C)	
4	162.2 (C)		162.2 (C)	
5	94.2 (CH)	5.97, s	94.2 (CH)	5.98, s
6	159.6 (C)		159.6 (C)	
7	209.5 (C)		209.5 (C)	
8	38.0 (CH)	3.89, sept. (6.7)	38.0 (CH)	3.89, sept. (6.7)
9	19.3 (CH_3_)	1.05, d (6.7)	19.3 (CH_3_)	1.05, d (6.7)
10	19.3 (CH_3_)	1.05, d (6.7)	19.3 (CH_3_)	1.05, d (6.7)
11	20.9 (CH_2_)	3.06, d (6.8)	20.9 (CH_2_)	3.07, dd (3.1, 6.4)
12	122.7 (CH)	5.11, t (6.8)	122.8 (CH)	5.09, t (6.7)
13	133.8 (C)		133.3 (C)	
14	16.1 (CH_3_)	1.66, s	16.1 (CH_3_)	1.66, s
15	36.4 (CH_2_)	1.82, m	35.2 (CH_2_)	1.82, m
		2.10, m		1.89, m
16	29.2 (CH_2_)	1.12, m	33.4 (CH_2_)	1.42, m
		1.52, m		
17	74.4 (CH)	3.14, dd (10.3, 4.6)	73.5 (CH)	3.78, t (7.2)
18	76.8 (C)		148.2 (C)	
19	21.6 (CH_3_)	1.01, s	17.6 (CH_3_)	1.59, s
20	19.6 (CH_3_)	0.94, s	109.8 (CH_2_)	4.67, s
21	48.6 (CH_3_)	3.03, s		4.79, s
HO-2		14.14, s		14.13, s
HO-4		10.26, s		10.26, s
HO-6		10.51, s		10.52, s

Hyperfaberol D (**4**), was obtained as a yellow gum, the molecular formula, C_20_H_28_O_5_, was established by HRESIMS data (*m/z* 347.1871 [M–H]^−^, calcd for 347.1864). Comparison of the 1D NMR data indicated that the structures of **4** and **3** were very similar to each other (Table 3). The difference lied in that compound **3** has one more methoxy group than **4**, meanwhile, the oxygenated quaternary carbon (*δ*_C_ 76.8, C-18) and Me-20 (*δ*_C_ 19.6) in **3** were also replaced by a terminal double bond (*δ*_C_ 148.2, C-18; and 109.8, C-20) in **4**, which could be confirmed by the ^1^H-^1^H COSY cross-peaks of H_2_-11/H-12 and H_2_-15/H_2_-16/H-17, and the HMBC correlations from H_3_-14 to C-12, C-13, and C-15, from H_2_-20 to C-17, C-18, and C-19, and from H_2_-11 to C-2, C-3, and C-4. In the ROESY spectrum, an NOE cross-peak between Me-14 and H_2_-11 was observed, indicating the *E* configuration of C-12/13 double bond of the moiety (Fig. 2). Thus, the structure of **4** was therefore established to be that show in Fig. 1.

Hyperfaberol E (**5**) gave the molecular formula of C_20_H_28_O_5_ by the HRESIMS *m/z* 347.1876 [M–H]^−^ (calcd for 347.1864). Its structure was elucidated to possess similar acylphloroglucinol scaffold with that of empetrikathiforin by detailed analysis of its MS and 1D NMR data [27]. The only difference lies in that the *sec*-butyl group in empetrikathiforin was replaced by an isopropyl in **5** [*δ*_H_ 3.86 (1H, sept., *J* = 6.7 Hz, H-8), 1.02 (3H each, d, *J* = 6.7 Hz, H-9/H-10); *δ*_C_ 38.1, C-8; 19.3, C-9/10] (Table 4), which was confirmed by the ^1^H-^1^H COSY cross-peaks of H-8/H_3_-9/H_3_-10 and the HMBC correlations of Me-9/Me-10 to C-7 and C-8 (Fig. 2).

**Table 4:** ^13^C (150 MHz) and ^1^H (600 MHz) NMR Data of **5** (in DMSO)

No.	*δ*_C_ (type)	*δ*_H_ (*J* in Hz)	No.	*δ*_C_ (type)	*δ*_H_ (*J* in Hz)
1	102.8 (C)		13	152.1 (C)	
2	164.1 (C)		14	108.1 (CH_2_)	4.61, s
3	104.2 (C)				4.78, s
4	163.0 (C)		15	26.0 (CH_2_)	2.03, m
5	94.5 (CH)	5.90, s	16	31.0 (CH_2_)	2.02, m
6	160.1 (C)		17	124.5 (CH)	5.06, m
7	209.5 (C)		18	130.6 (C)	
8	38.1 (CH)	3.86, sept. (6.7)	19	25.5 (CH_3_)	1.59, s
9	19.3 (CH_3_)	1.02, d (6.7)	20	17.5 (CH_3_)	1.52, s
10	19.3 (CH_3_)	1.02, d (6.7)	HO-2		13.90, s
11	29.3 (CH_2_)	2.66, dd (13.5, 5.3)2.53, m	HO-4		10.26, s
12	73.5 (CH)	4.14, t (6.3)	HO-6		10.78, s

Hyperfaberol F (**6**) was isolated as an amorphous powder. Its molecular formula of C_13_H_14_O_4_ was assigned on the basis of its ^13^C NMR data and the observed [M–H]^−^ ion at m/z 233.0823 (calcd for C_13_H_13_O_4_, 233.0819). The IR absorptions implied the presence of hydroxy (3417 cm^−1^) and conjugated carbonyl (1622 cm^−1^) groups. The ^1^H NMR revealed the presence of two aromatic protons at *δ*_H_ 6.14 (1H, d, *J* = 1.6 Hz) and 5.97 (1H, d, *J* = 1.6 Hz), and a singlet olefinic proton at *δ*_H_ 5.94 (1H, s), as well as a *sec*-butyl group at *δ*_H_ 2.49 (1H, m), 1.03 (3H, d, *J* = 6.9 Hz), 1.38 and 1.50 (2H, each m), and 0.68 (3H, s). The ^13^C NMR spectrum exhibited 13 signals, including nine sp^2^ carbons (one carbonyl, six aromatic carbons, and two olefinic carbons) and four sp^3^ carbons (two methyls, one methylene, and one methine) (Table 5). The nine downfield sp^2^ signals indicated that **6** possessed a chromone skeleton, while the four signals for sp^3^ carbons relatively upfield confirmed the presence of the *sec*-butyl group observed in the ^1^H NMR spectrum. The 1D NMR data of **6** was similar to those of 5, 7-dihydroxy-2-isopropylchromon (**7**) [17], the difference lies in that the isopropyl in **7** was substituted by a *sec*-butyl group (*δ*_C_ 39.4, C-9; 17.5, C-10; 26.9, C-11; and 11.4, C-12) in **6**, which can be proved by the correlations from H-9 to C-2 in the HMBC spectrum, as well as the long linkage of H-9/H_3_-10/H_2_-11/H_3_-12 in the ^1^H-^1^H COSY spectrum (Fig. 2). Thus, the structrue of **6** was assigned as shown.

**Table 5:** ^13^C (150 MHz) and ^1^H (600 MHz) NMR Data of **6** (in DMSO)

No.	*δ*_C_ (type)	*δ*_H_ (*J* in Hz)	No.	*δ*_C_ (type)	*δ*_H_ (*J* in Hz)
**2**	173.6 (C)		8	98.9 (CH)	5.97, d (1.6)
**3**	106.4 (CH)	5.94, s	8a	161.5 (C)	
**4**	181.7 (C)		9	39.4 (CH)	2.49, m
**4a**	103.4 (C)		10	17.5 (CH_3_)	1.03, d (6.9)
**5**	157.9 (C)		11	26.9 (CH_2_)	1.38, m
**6**	93.8 (CH)	6.14, d (1.6)			1.50, m
**7**	164.9 (C)		12	11.4 (CH_3_)	0.68, s

**Fig. 3:**
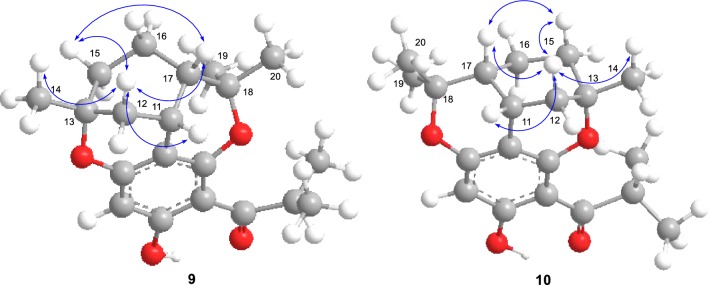
Key NOESY correlations of **9** and **10**

Empetrifranzinan A/B (**9**/**10**) have been previously described from *Hypericum empetrifolium* [18]. However, the relative configurations of the two compounds were not given in previous literature, and which are demonstrated herein. In the ROESY spectrum of **9**, the NOE correlations of H-11/H-12a, H-12a/H-15b/H-17, and H-12a/H_3_-14 suggested the *β*-orientation of H-11, Me-14, and H-17. The same relative configuration of **10** was also established as shown in Fig. 3.

The inhibitory activities of the isolates were examined against the five human tumor cell lines (ECA-109, PANC-1, BIU-87, and BEL-7402) with taxol as the positive control (IC_50_ values of 1.2, 3.5, 2.1 and 1.9 μM, respectively) [28]. Compound **1** showed cytotoxicity against the human esophageal cancer cell line (ECA-109) with an IC_50_ value of 12.8 μM, while compound **11** exhibited activity against the pancreatic tumor cell line (PANC-1) with an IC_50_ value of 12.0 μM. Additionally, other isolates showed no activities against the five human tumor cell lines with IC_50_ values > 30 μM.

## Experimental

### General Experimental Procedures

Optical rotations were measured on a JASCO P-1020 polarimeter. UV spectra were recorded on a Shimadzu UV-2401PC spectrometer. IR spectra were recorded on a Bruker FT-IR Tensor-27 infrared spectrophotometer with KBr disks. 1D and 2D NMR spectra were recorded on a Bruker DRX-600 spectrometer using TMS as an internal standard. The chemical shifts (*δ*) were expressed in ppm with reference to the solvent signals. ESIMS and HR-ESIMS analysis were carried out on Waters Xevo TQS and Agilent G6230 TOF mass spectrometers, respectively. Preparative and semi-preparative HPLC were performed on Waters 1525 HPLC (column: SunFire OBD-C_18_, 19 × 250 mm) and Agilent 1100 HPLC (column: Zorbax SB-C_18_, 9.4 × 250 mm), respectively. Silica gel (100–200 and 200–300 mesh, Qingdao Marine Chemical Co., Ltd., People’s Republic of China), and MCI gel (75–150 μm, Mitsubishi Chemical Corporation, Tokyo, Japan) were used for column chromatography. Fractions were monitored by TLC (GF 254, Qingdao Marine Chemical Co., Ltd.), and spots were visualized by heating silica gel plates immersed in H_2_SO_4_ in ethanol.

### Plant Material

The whole plants of *H. faberi* were collected in Yiliang County, Zhaotong City, Yunnan Province, China. It was identified by Dr. Yong-Zeng Zhang in Kunming Institute of Botany. A voucher specimen (no. 201707H01) has been deposited at the Kunming Institute of Botany.

### Extraction and Isolation

The dried whole plants of *H. faberi* (15.0 kg) were powdered and percolated with MeOH at room temperature and filtered. The filtrate was evaporated in vacuo to be concentrated. The crude extract (3.2 kg) was subjected to a silica gel column chromatography (CC) using elution of successive CHCl_3_ and EtOAc to give a non-polar fraction (163.0 g) and a polar fraction (192 g). The CHCl_3_ fraction was separated by an MCI-gel CC (MeOH–H_2_O, 70–100%) to yield six fractions (Fr. 1–6, polarity from small to large). Fr. 3 (10.2 g) was separated by a Rp-18 column (49 × 460 mm, MeOH–H_2_O, 80–100%) to obtain eleven subfractions (Fr. 3-1 to Fr. 3-11) on the basis of TLC analysis. Fr. 3-7 (226.1 mg) was fractioned by preparative HPLC (MeOH–H_2_O, 88%) to afford Fr. 3-7-1, which was further purified by semipreparative HPLC (MeOH–H_2_O, 85%) to give the **11** (5.6 mg). Fr. 3-8 (271.7 mg) was fractioned by preparative HPLC (MeOH–H_2_O, 86%) to afford Fr. 3-8-1, which was further separated by semipreparative HPLC (MeOH–H_2_O, 80%) to obtain compound **1** (4.7 mg). Fr. 3-10 (184.2 mg) was purified by preparative HPLC (MeOH–H_2_O, 80%) to yield compound **9** (8.4 mg) and **10** (5.0 mg). Fr. 4 (11.5 g) was separated by a Rp-18 column (MeOH–H_2_O, 70–100%) to obtain thirteen subfractions (Fr. 4–1 to Fr. 4–13). Fr. 4–7 (394.6 mg) was fractioned by preparative HPLC (MeOH–H_2_O, 93%) to afford Fr. 4–7–1, which was then purified by semipreparative HPLC (MeOH–H_2_O, 90%) to afford the **2** (1.3 mg).

The EtOAc fraction (192 g) was separated by an MCI-gel CC (MeOH–H_2_O, 45–90%) to obtain five fractions (Fr. E1–E5, polarity from small to large). Fr. E1 (25.1 g) was separated by silica gel CC (petroleum ether/EtOAc, from 400:1 to 0:1) to give eight subfractions, Fr. E1–1 to Fr. E1–8. Fr. E1–4 (603.1 mg) was purified by repeated preparative HPLC (MeOH/H_2_O: 80%) to yield the **3** (2.6 mg). Fr. E2 (11.7 g) was separated by silica gel CC (petroleum ether/EtOAc, from 50:1 to 0:1) to give eleven subfractions, Fr. E2–1 to Fr. E2–11. Fr. E2–3 (142.3 mg) was further purified by preparative HPLC (MeOH/H_2_O: 75%) to yield the **5** (1.8 mg). Fr. E2–4 (1.4352 g) was further isolated sequentially using Sephadex LH-20 (Acetone), preparative HPLC (MeOH/H_2_O: 70%) to yield the **12** (1.7 mg). Fr. E3 (10.5 g) was separated by silica gel CC (petroleum ether/EtOAc, from 25:1 to 0:1) to obtain ten subfractions, Fr. E3–1 to Fr. E3–10. Fr. E3–3 (533.5 mg), Fr. E3–4 (392.1 mg), and Fr. E3–6 (1.2 g) was separated by Sephadex LH-20 (Acetone), and purified by prep-HPLC (MeOH/H_2_O: 70%) to afford compounds **6** (1.8 mg), **7** (2.3 mg), **8** (2.1 mg), **13** (1.5 mg). Fr. E3–8 (562.7 mg) was separated by silica gel CC (petroleum ether/EtOAc, from 10:1 to 0:1) to give eight subfractions, Fr. E3–8–1 to Fr. E3–8–8. Fr. E3–8–2 (405.2 mg) was further subjected to a silica gel CC (CDCl_3_/MeOH, from 400:1 to 0:1) to give Fr. E3–8–2–1 to Fr. E3–8–2–8. Fr. E3–8–2–4 (62.4 mg) was further purified by semipreparative HPLC (MeOH/H_2_O: 70%) to yield the **4** (3.3 mg).

#### Hyperfaberol A (**1**)

Yellow amorphous powder; [*α*]_D_^22^ +19 (*c* 0.12, MeOH); UV (MeOH) *λ*_max_ (log *ε*): 385 (3.74), 333 (4.25), 267 (4.36), 245 (4.33), 203 (4.52) nm; IR (KBr) *ν*_max_: 3423, 2968, 2925,1634, 1574, 1454 cm^−1^; ^1^H and ^13^C NMR data, see Table 1; ESIMS *m*/*z* 461 [M–H]^−^; HR-ESIMS *m/z* 461.1979 [M–H]^−^ (calcd for C_28_H_29_O_6_, 461.1970).

#### Hyperfaberol B (**2**)

Yellow amorphous powder; [*α*]_D_^20^ +6 (*c* 0.13, MeOH); UV (MeOH) *λ*_max_ (log *ε*): 323 (4.4), 283 (4.02), 248 (4.66), 214 (4.40) nm; IR (KBr) *ν*_max_: 3370, 3083, 2925, 2854, 2731, 1664, 1613, 1518, 1403, 1374, 1228, 1161, 1032 cm^−1^; ^1^H and ^13^C NMR data, see Table 2; ESIMS *m*/*z* 339 [M–H]^−^; HR-ESIMS *m/z* 339.0881 [M–H]^−^ (calcd for C_19_H_15_O_6_, 339.0874).

#### Hyperfaberol C (**3**)

Yellow gum; [*α*]_D_^20^−34 (*c* 0.12, MeOH); UV (MeOH) λ_max_ (log ε) 291 (4.16), 197 (4.31) nm; IR (KBr) *ν*_max_ 3417, 2975, 2932, 2874, 2125, 1622, 1519, 1431, 1383, 1354, 1301 cm^−1^; ^1^H and ^13^C NMR data, see Table 3; ESIMS *m*/*z* 379 [M–H]^−^; HR-ESIMS m/z 379.2136 [M–H]^−^ (calcd for C_21_H_31_O_6_, 379.2126).

#### Hyperfaberol D (**4**)

Yellow gum; [*α*]_D_^20^−23 (*c* 0.16, MeOH); UV (MeOH) λ_max_ (log ε) 291 (3.86), 196 (4.06) nm; IR (KBr) *ν*_max_ 3427, 2969, 2929, 2876, 2730, 1602, 1519, 1384, 1296, 1140, 1034 cm^−1^; ^1^H and ^13^C NMR data, see Table 3; ESIMS *m*/*z* 347 [M–H]^−^; HR-ESIMS m/z 347.1871 (calcd for C_20_H_27_O_5_, 347.1864).

#### Hyperfaberol E (**5**)

Yellow gum; [*α*]_D_^20^−19 (*c* 0.20, MeOH); UV (MeOH) λ_max_ (log ε) 290 (3.23), 228 (3.15), 197 (3.39) nm; IR (KBr) *ν*_max_ 3428, 2966, 2932, 2873, 1623, 1518, 1433, 1354, 1238, 1171, 1023 cm^−1^; ^1^H and ^13^C NMR data, see Table 4; ESIMS *m*/*z* 347 [M–H]^−^; HR-ESIMS m/z 347.1876 [M–H]^−^ (calcd for C_20_H_27_O_5_, 347.1864).

#### Hyperfaberol F (**6**)

Yellow powder; [*α*]_D_^20^−12 (*c* 0.10, MeOH); UV (MeOH) λ_max_ (log ε) 294 (3.82), 250 (4.19), 227 (4.14), 202 (4.31) nm; IR (KBr) *ν*_max_ 3417, 3093, 3071, 2967, 2934, 2878, 2726, 2633, 1622, 1107, 1011 cm^−1^; ^1^H and ^13^C NMR data, see Table 5; ESIMS *m*/*z* 233 [M–H]^−^; HR-ESIMS m/z 233.0823 [M–H]^−^ (calcd for C_13_H_13_O_4_, 233.0819).

#### Cytotoxcity Assay

The cytotoxic assay with taxol as a positive control was conducted by MTT method in 96-well plates reported previously (Biological Assay, Supporting Information) [7].

## Electronic supplementary material

Below is the link to the electronic supplementary material.

Supplementary material 1 (DOCX 6453 kb)
