# Correction to: Diverse Polyphenols from *Hypericum faberi*

**DOI:** 10.1007/s13659-019-00216-1

**Published:** 2019-08-05

**Authors:** Xin-Wen Zhang, Yan-Song Ye, Fan Xia, Xing-Wei Yang, Gang Xu

**Affiliations:** 1 State Key Laboratory of Phytochemistry and Plant Resources in West China, Kunming Institute of Botany, Chinese Academy of Sciences, Yunnan Key Laboratory of Natural Medicinal Chemistry, Kunming, 650201 People’s Republic of China; 2 University of Chinese Academy of Sciences, Beijing, 100049 People’s Republic of China

## Correction to: Natural Products and Bioprospecting (2019) 9:215–221 10.1007/s13659-019-0206-1

In the original publication the corresponding author appeared incorrectly as Xin-Wen Zhang.

The corrected text is given below:

The corresponding author of the article is Gang Xu.

