# Supplementary material for: Diverse Polyphenols from Hypericum faberi
Source: Nat Prod Bioprospect. 2019 May 9;9(3):215–21. doi: 10.1007/s13659-019-0206-1 (PMC6538727; doi:10.1007/s13659-019-0206-1)
Supplement: Supplementary file 1 — Supplementary material 1 (DOCX 6453 kb) [file 13659_2019_206_MOESM1_ESM.docx]

**Diverse Polyphenols from *Hypericum faberi***

**Xin-Wen Zhang, Yan-Song Ye, Fan Xia, Xing-Wei Yang, and Gang Xu**

Supplementary material

Table of Contents

- Biological assay (Page S2)
- Figures S1–S45: The Original NMR and MS spectra of the new compounds (Page S3–S27)

**Biological assay**

Colorimetric assays were performed to evaluate compound activity. The following human tumor cell lines were used: ECA-109 esophageal cancer cell line, PANC-1 pancreatic cell line, BIU-87 bladder cancer cell line, and BEL-7402 hepatoma cell line. All cells were cultured in RPMI-1640 or DMEM medium (Hyclone, Logan, UT), supplemented with 10% fetal bovine serum (Hyclone) at 37 °C in a humidified atmosphere with 5% CO_2_. Cell viability was assessed by conducting colorimetric measurements of the amount of insoluble formazan formed in living cells based on the reduction of 3-(4,5-dimethylthiazol-2-yl)-2,5-diphenyltetrazolium bromide (MTT). Briefly, 100 *μ*L adherent cells were seeded into each well of a 96-well cell culture plate and allowed to adhere for 12 h before test compound addition, while suspended cells were seeded just before this step, both with initial density of 1 × 10^5^ cells/mL in 100 *μ*L of medium. Each tumor cell line was exposed to the test compound at various concentrations in triplicate for 48 h, with taxol as positive control. After the incubation, MTT (100 *μ*g) was added to each well, and the incubation continued for 4 h at 37 °C. The cells were lysed with 100 *μ*L of 20% SDS-50% DMF after removal of 100 *μ*L of medium. The optical density of the lysate was measured at 595 nm in a 96-well microtiter plate reader (Bio-Rad 680). The IC_50_ value of each compound was calculated by Reed and Muench’s method.


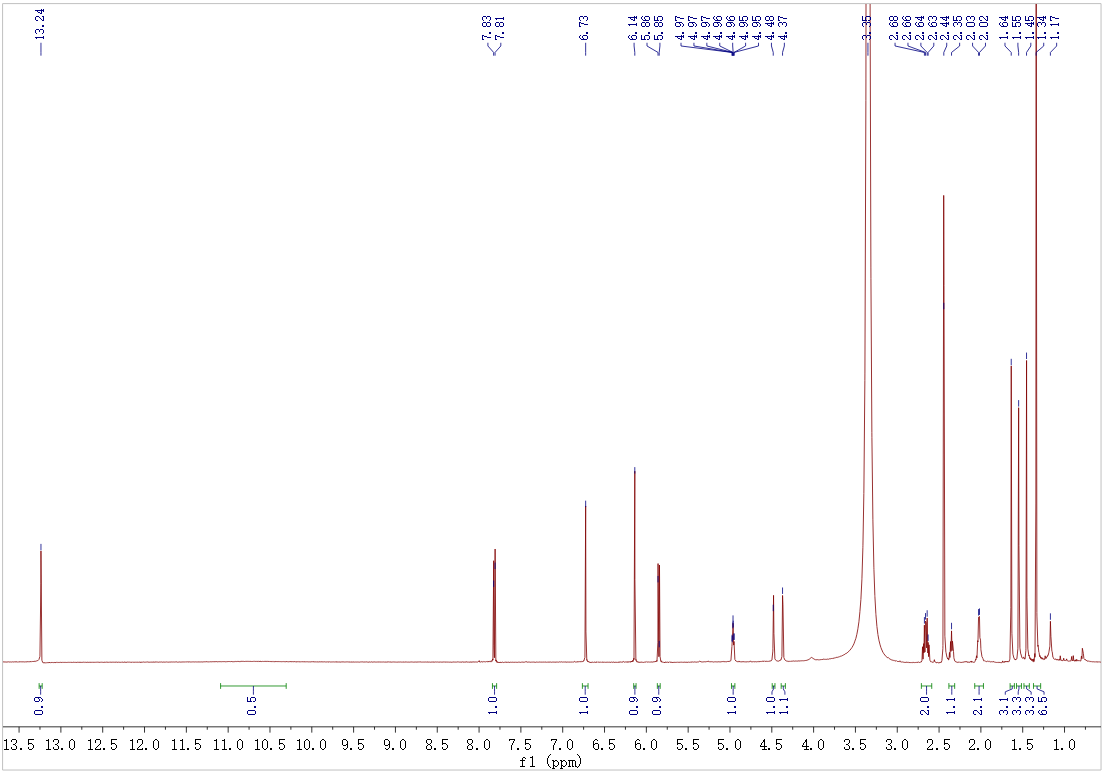


**Fig. S1** ^1^H (in DMSO-*d_6_*) spectrum of hyperfaberol A (**1**).


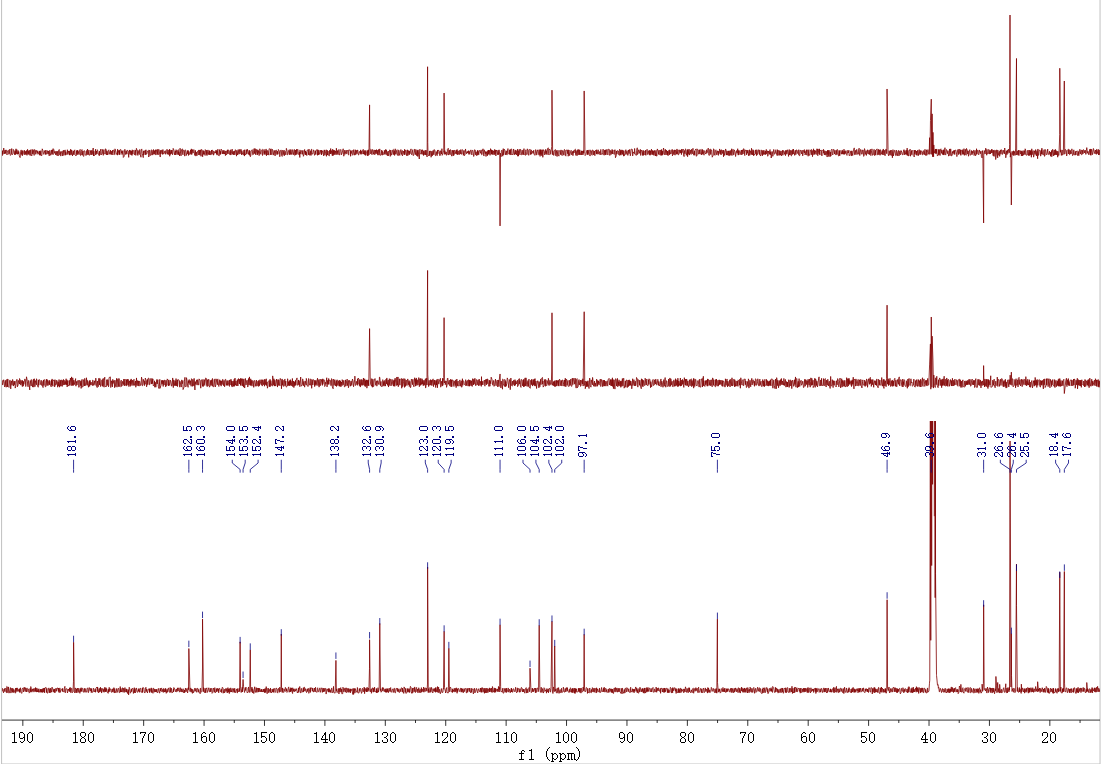


**Fig. S2** ^13^C and DEPT (in DMSO-*d_6_*) spectra of hyperfaberol A (**1**).
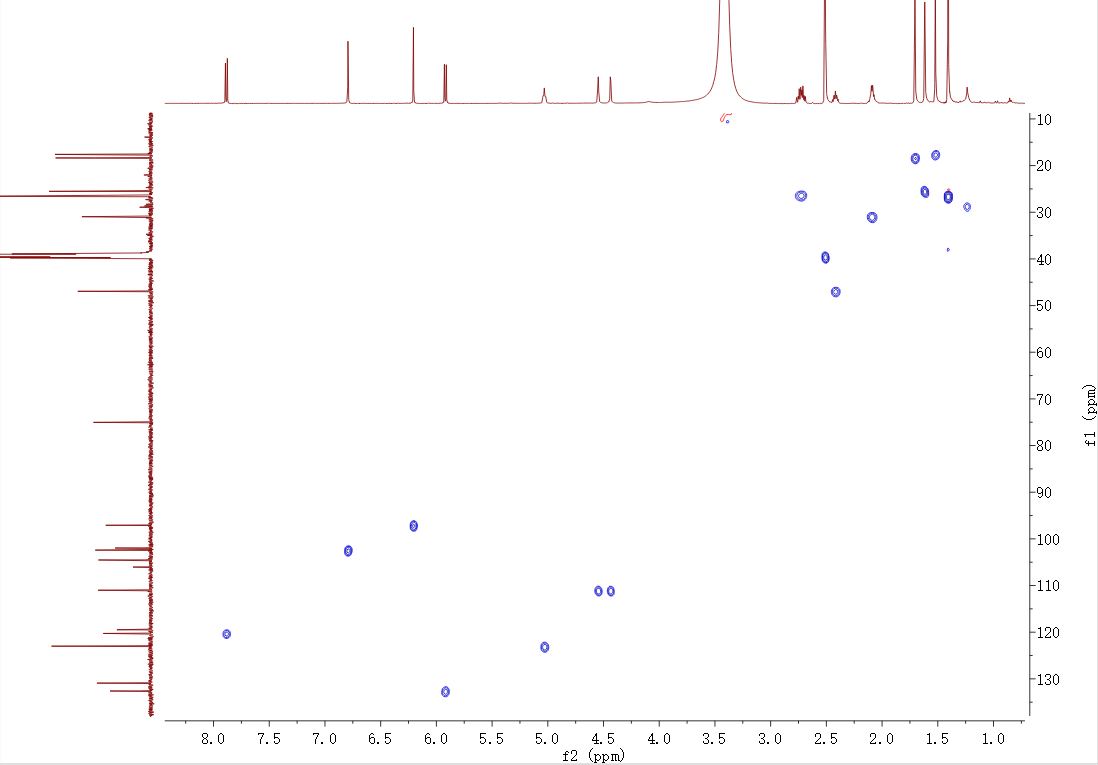


**Fig. S3** HSQC spectrum of hyperfaberol A (**1**).


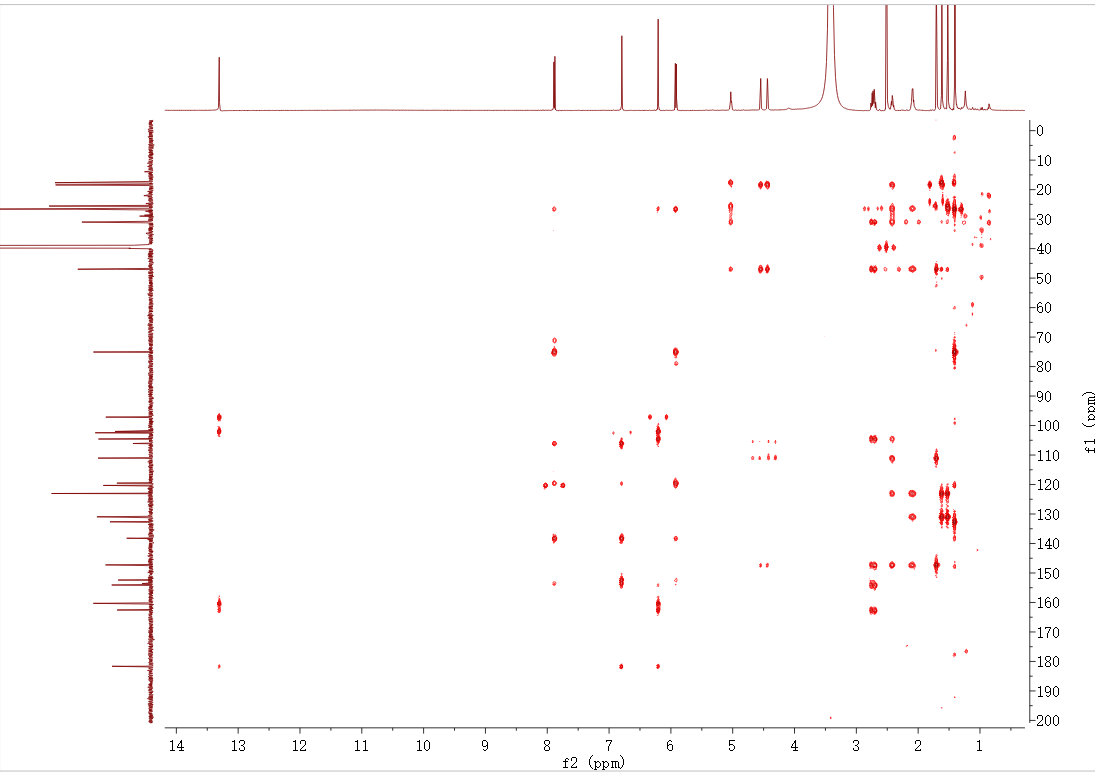


**Fig. S4** HMBC spectrum of hyperfaberol A (**1**).


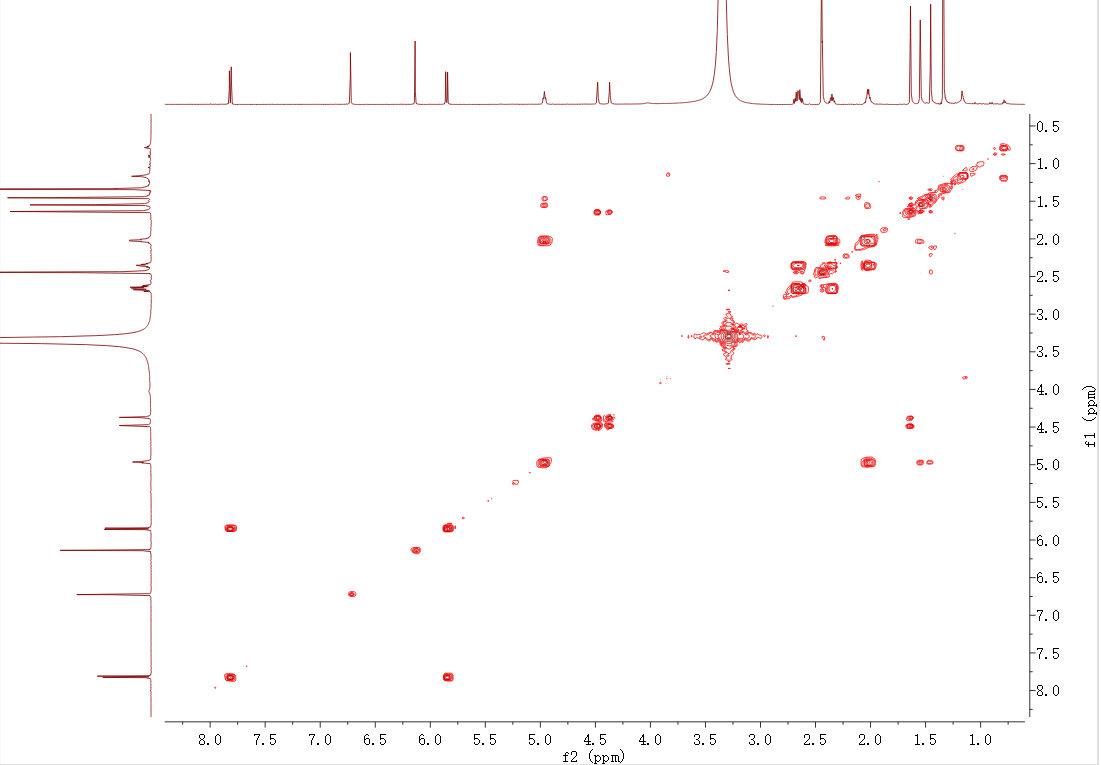


**Fig. S5** ^1^H–^1^H COSY spectrum of hyperfaberol A (**1**).


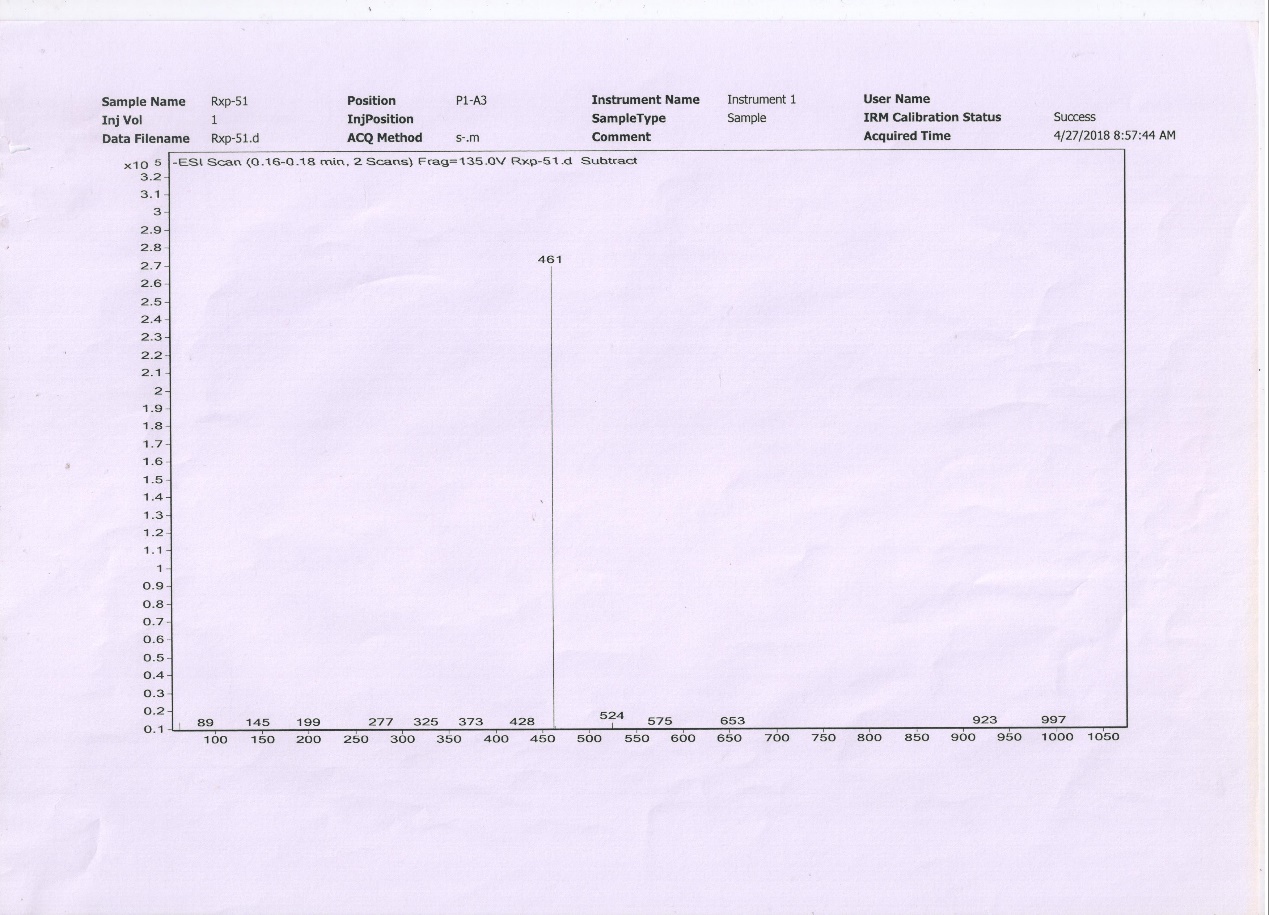


**Fig. S6** ESIMS spectrum of hyperfaberol A (**1**).


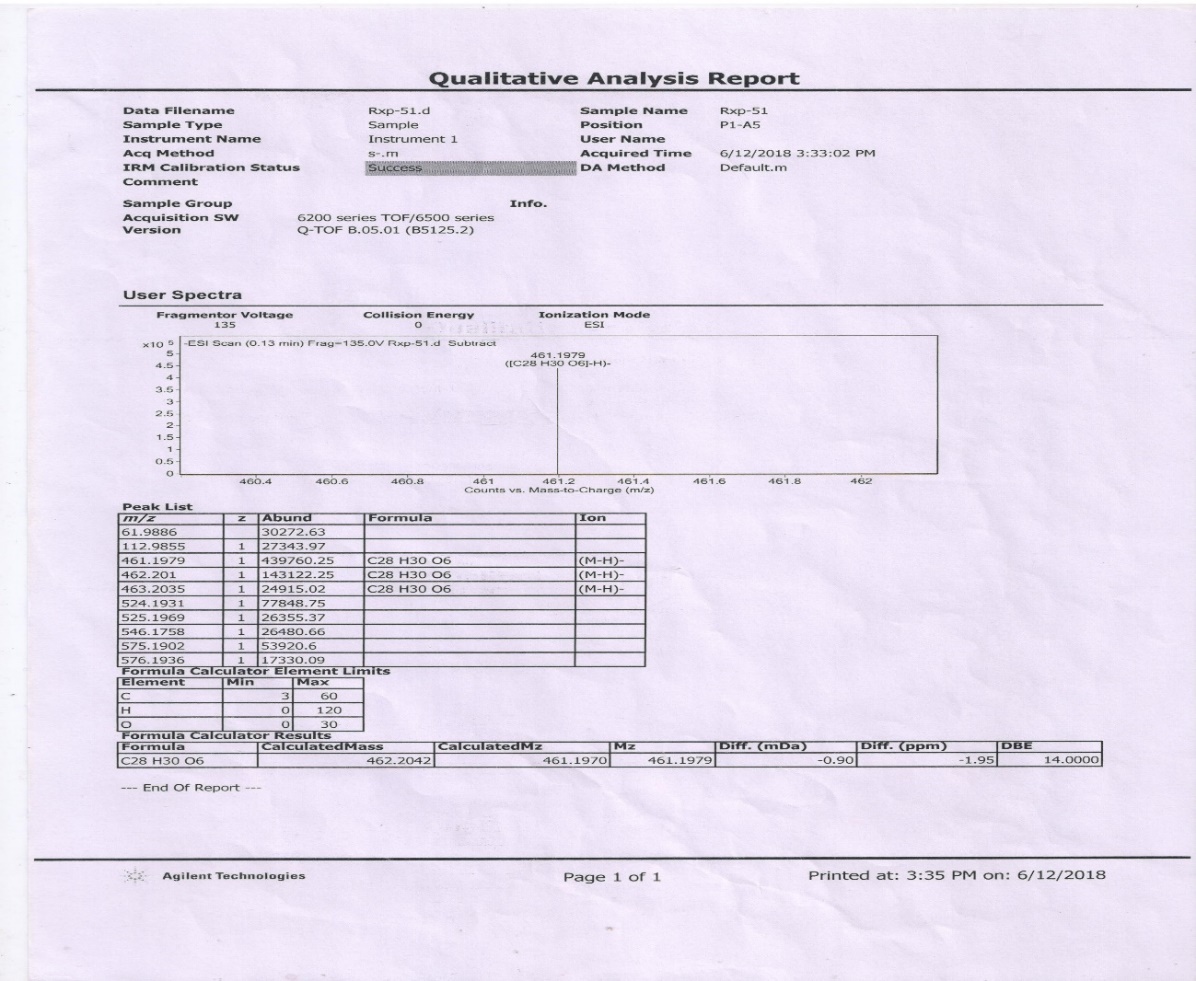


**Fig. S7** HRESIMS spectrum of hyperfaberol A (**1**).


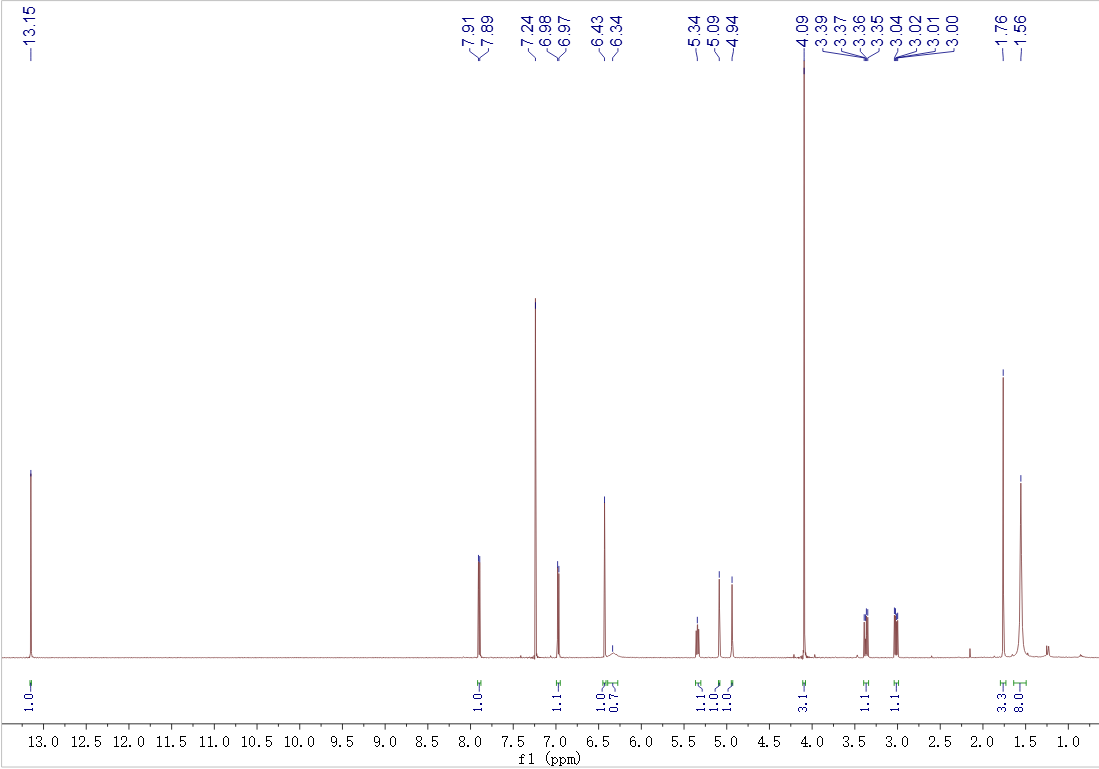


**Fig. S8** ^1^H (in CDCl_3_) spectrum of hyperfaberol B (**2**).


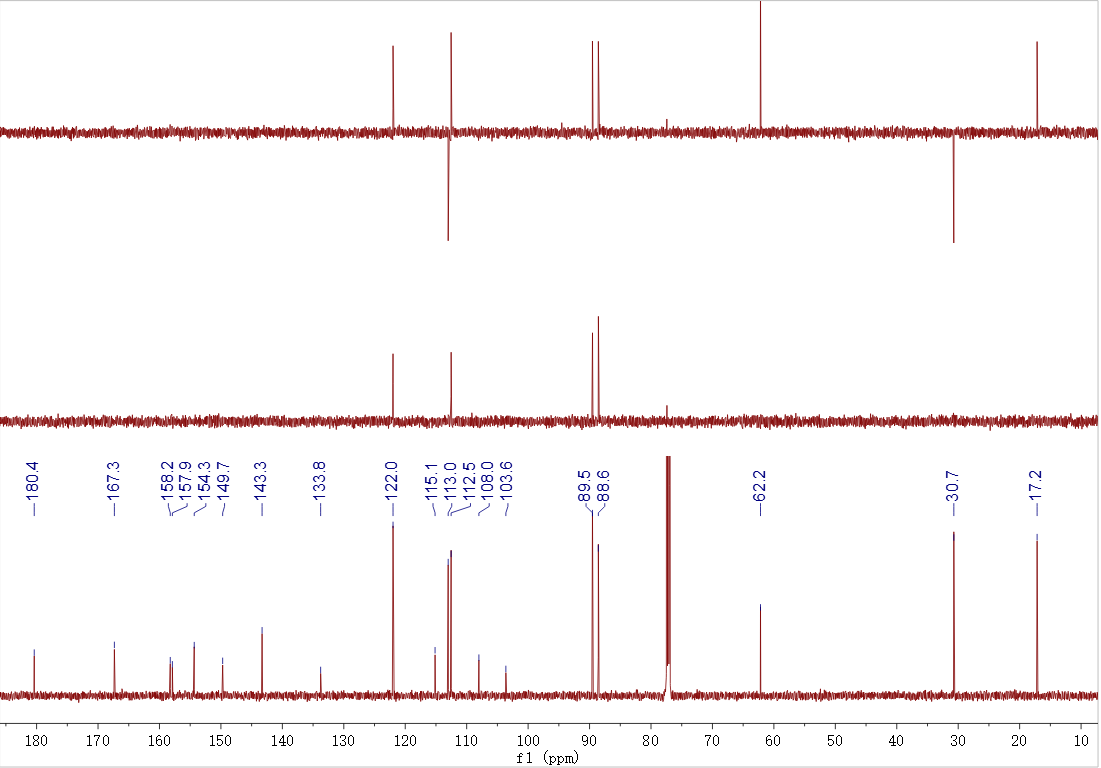


**Fig. S9** ^13^C and DEPT (in CDCl_3_) spectra of hyperfaberol B (**2**).


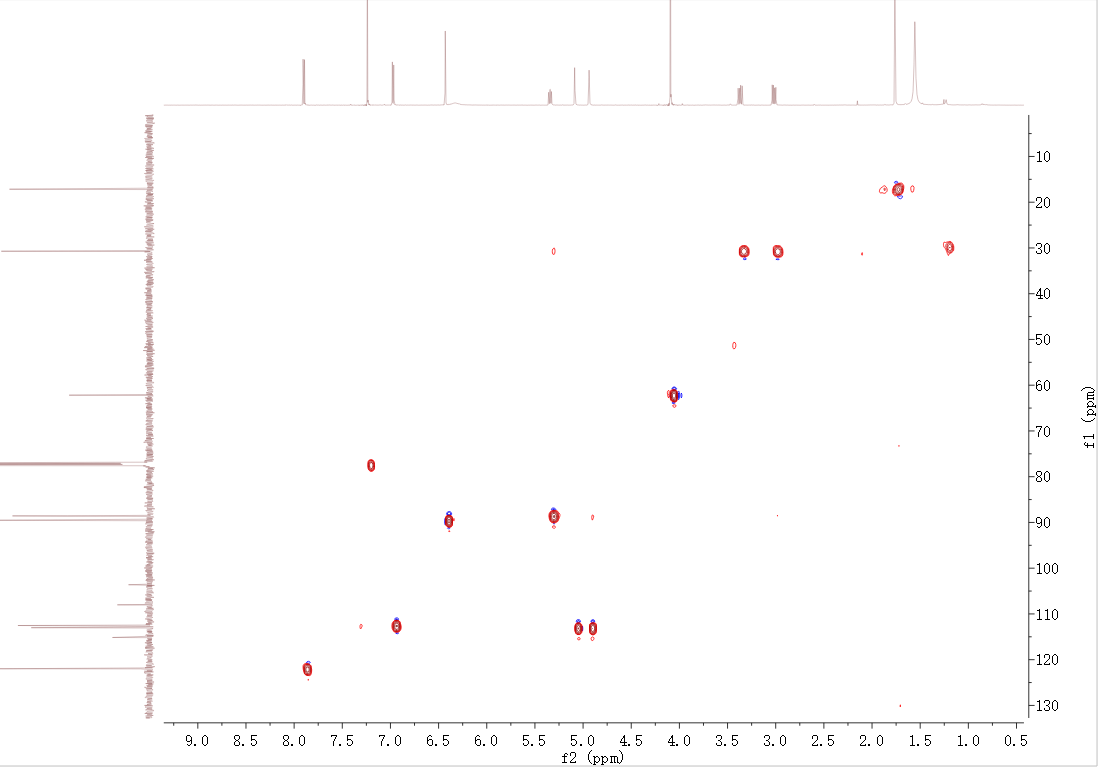


**Fig. S10** HSQC spectrum of hyperfaberol B (**2**).


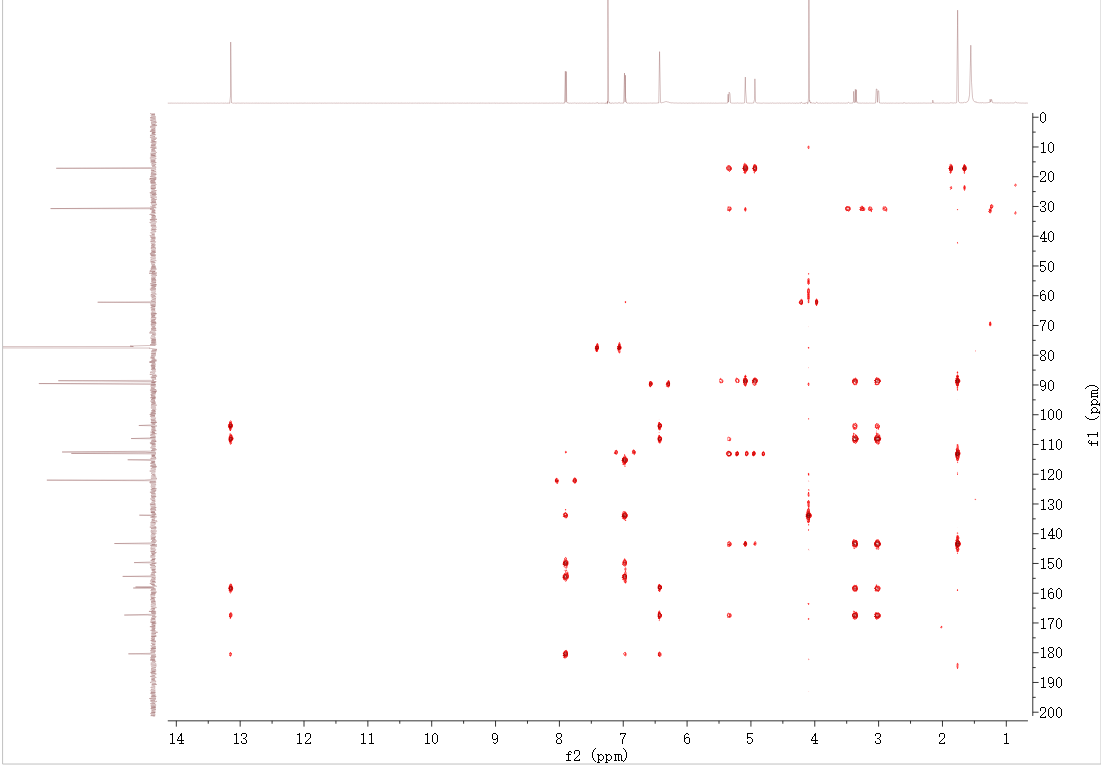


**Fig. S11** HMBC spectrum of hyperfaberol B (**2**).


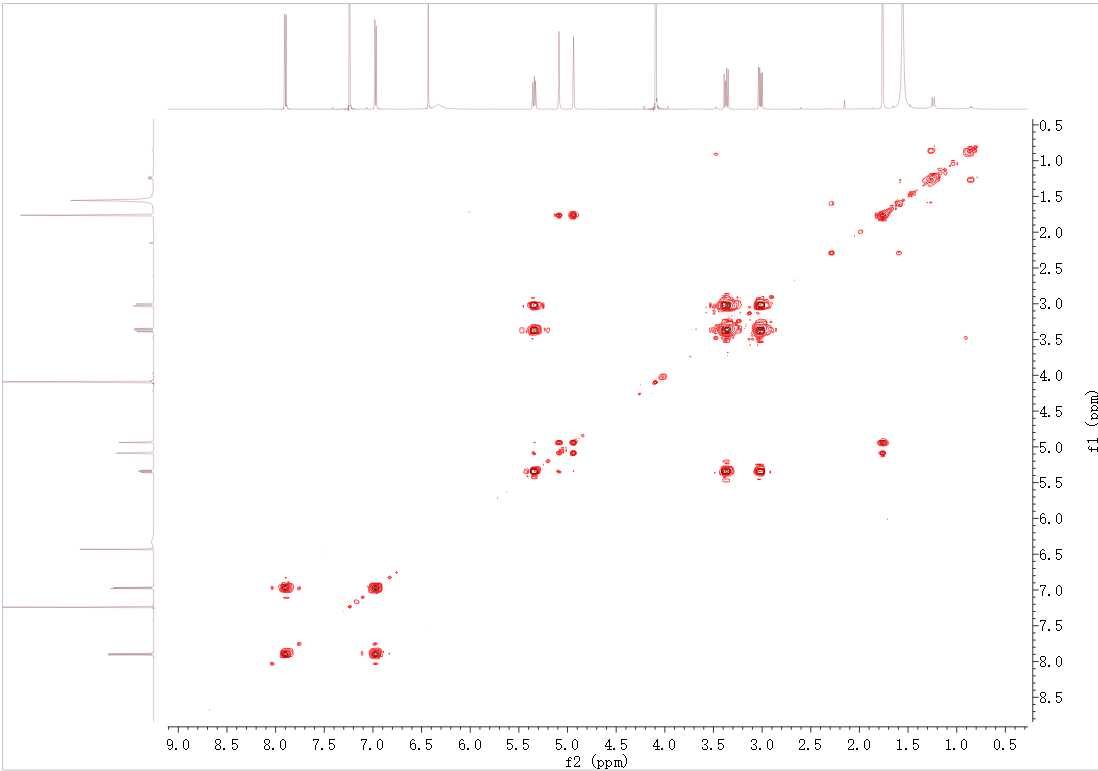


**Fig. S12** ^1^H–^1^H COSY spectrum of hyperfaberol B (**2**).


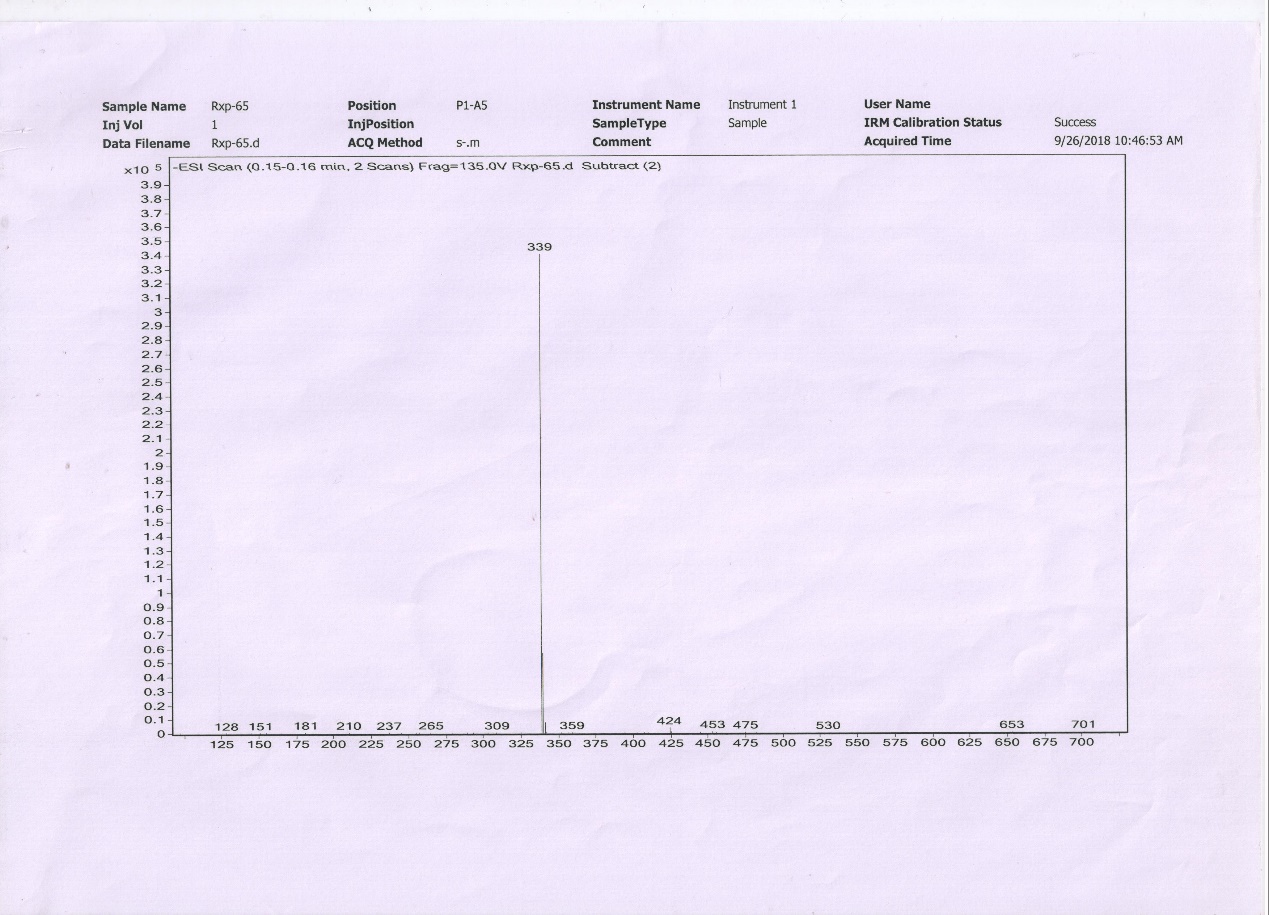


**Fig. S13** ESIMS spectrum of hyperfaberol B (**2**).


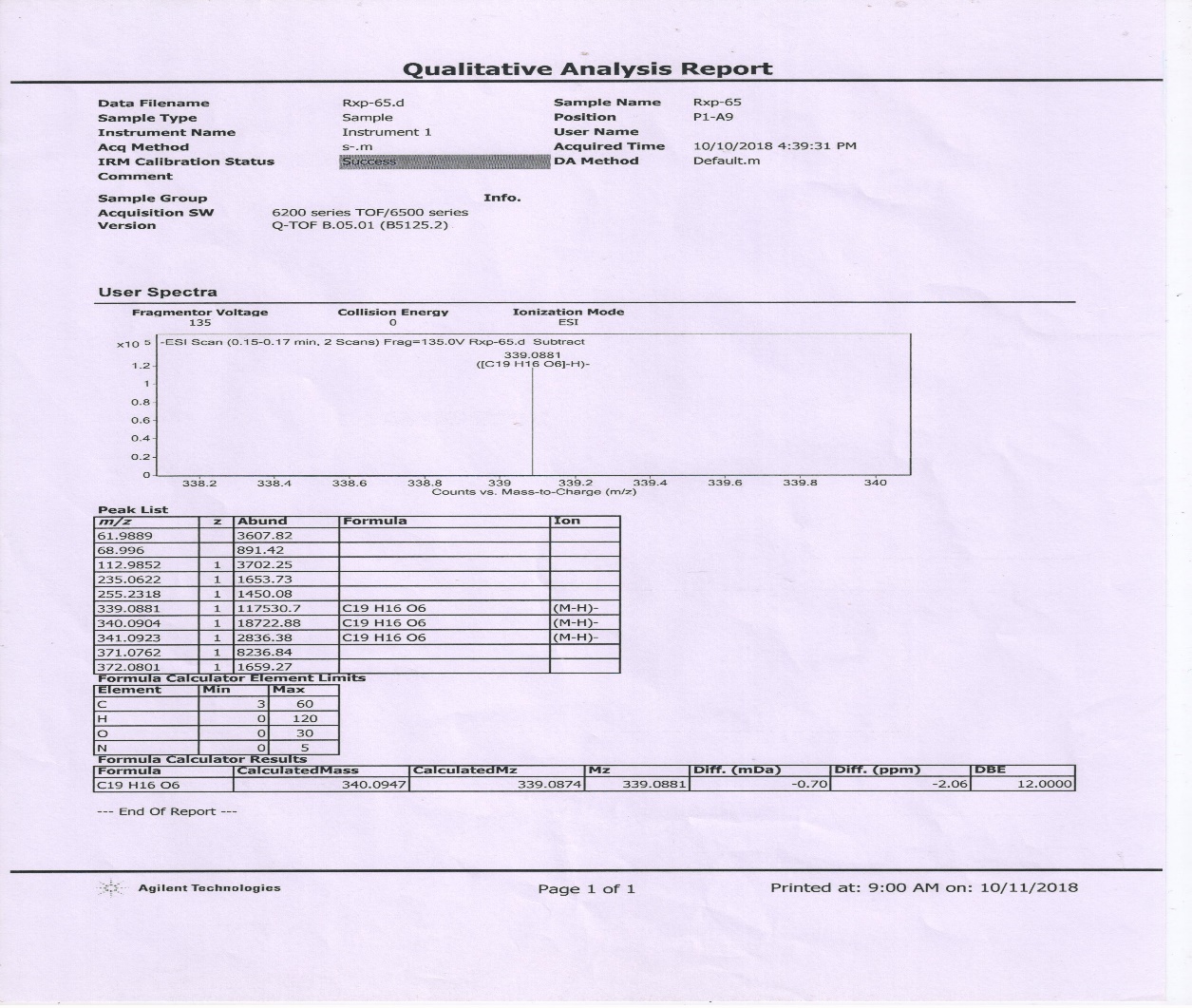


**Fig. S14** HRESIMS spectrum of hyperfaberol B (**2**).


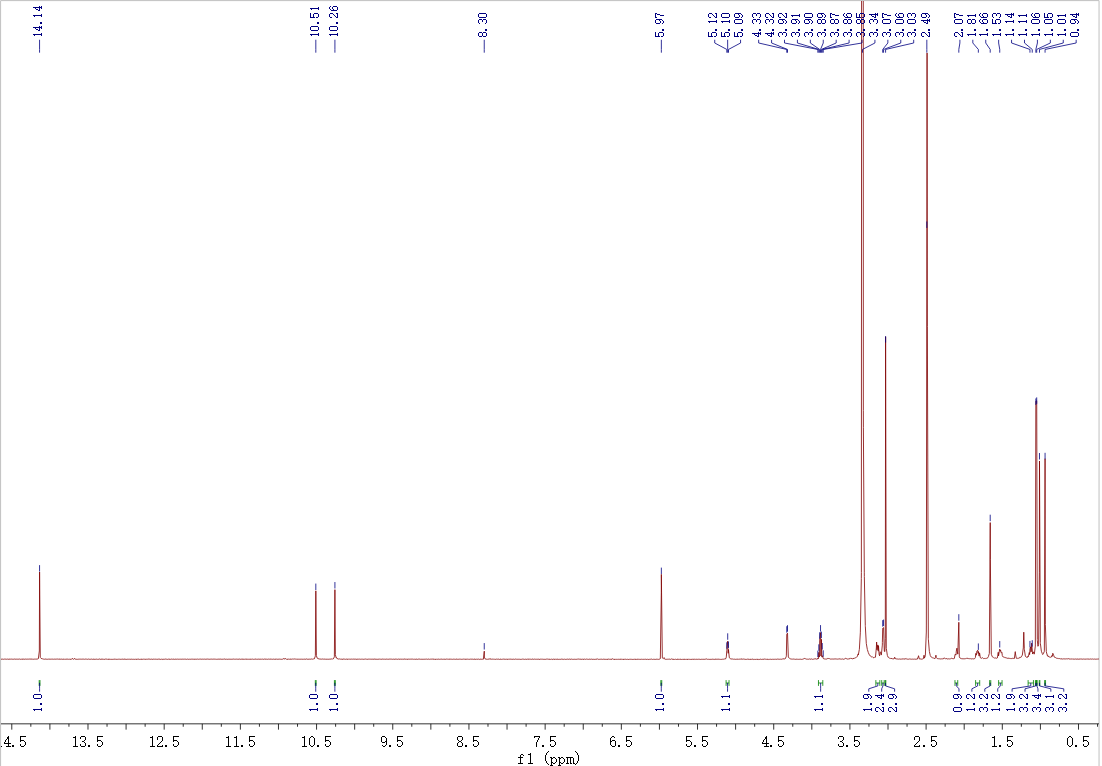


**Fig. S15** ^1^H (in DMSO-*d_6_*) spectrum of hyperfaberol C (**3)**.


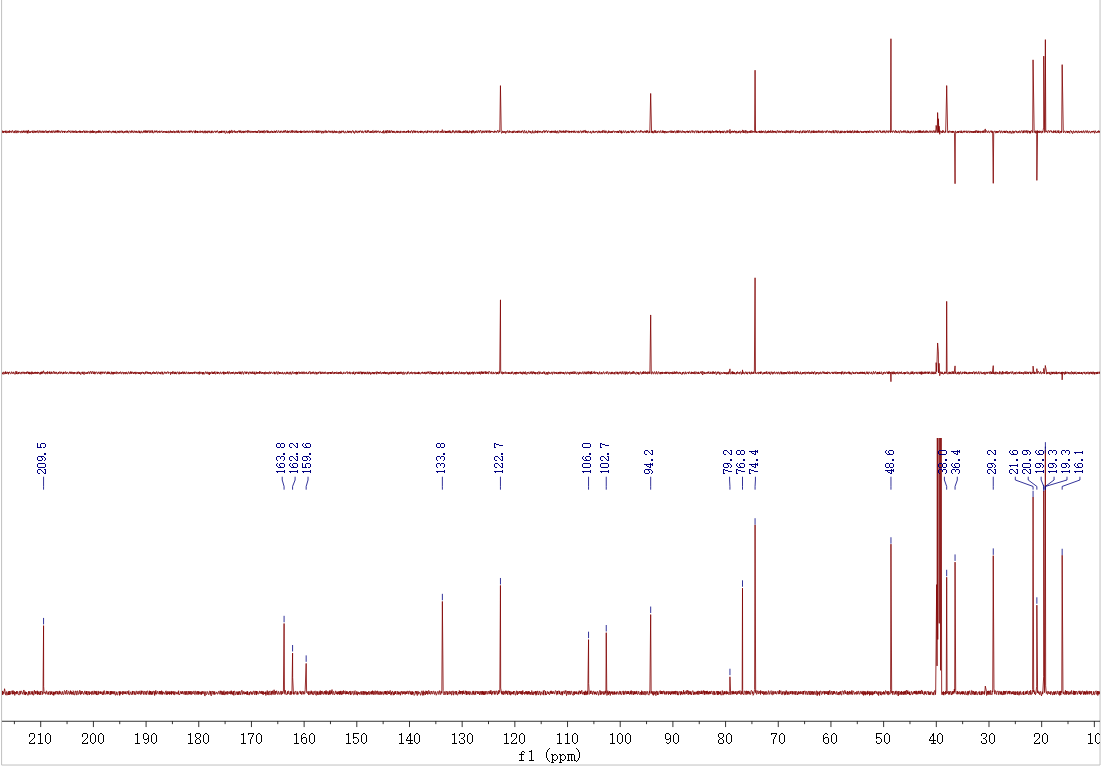


**Fig. S16** ^13^C and DEPT (in DMSO-*d_6_*) spectrum of hyperfaberol C (**3)**.


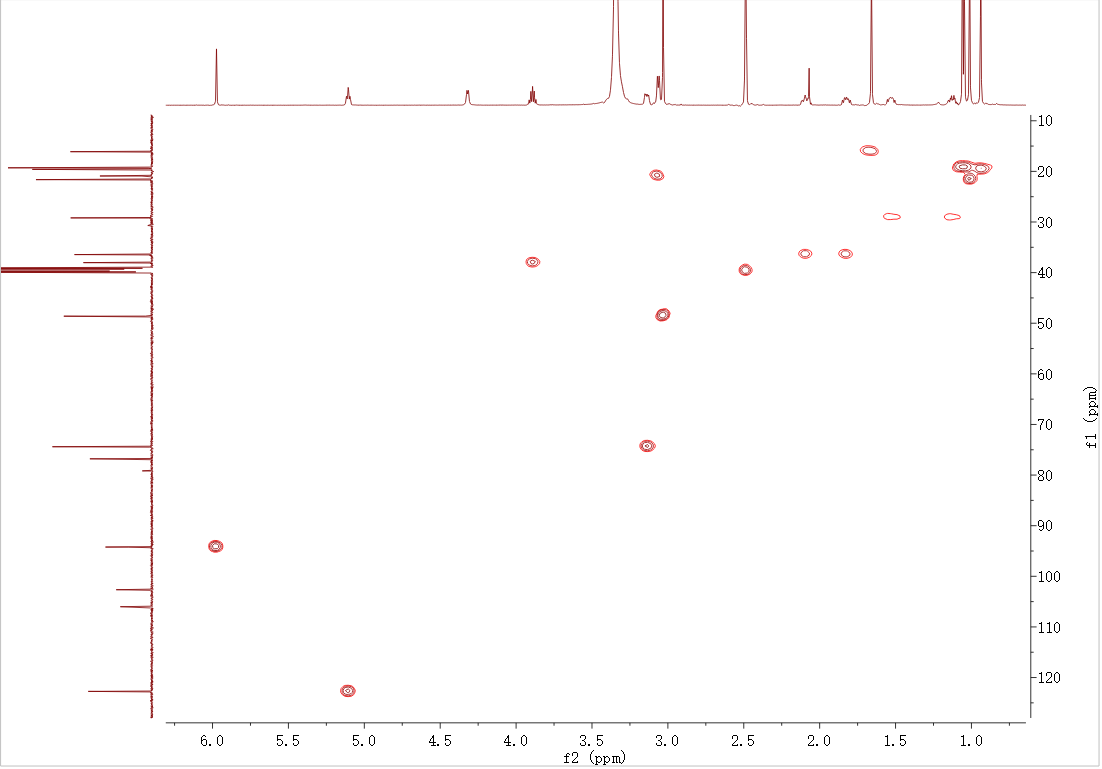


**Fig. S17** HSQC spectrum of hyperfaberol C (**3)**.


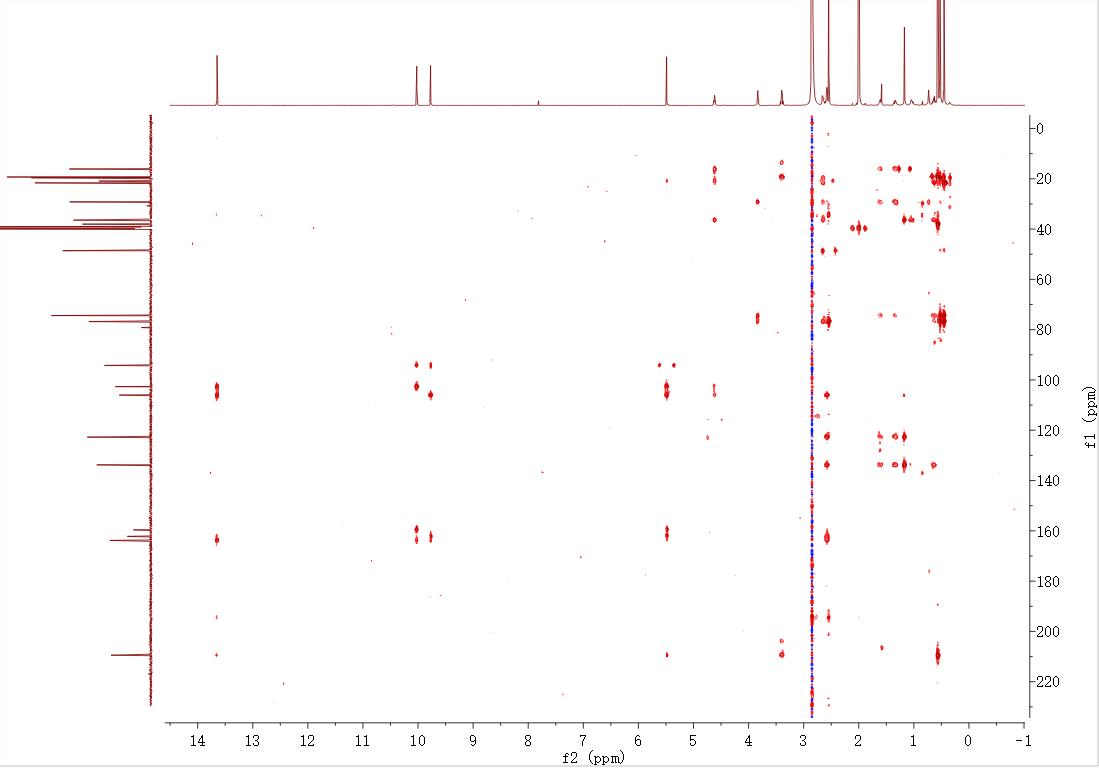


**Fig. S18** HMBC spectrum of hyperfaberol C (**3)**.


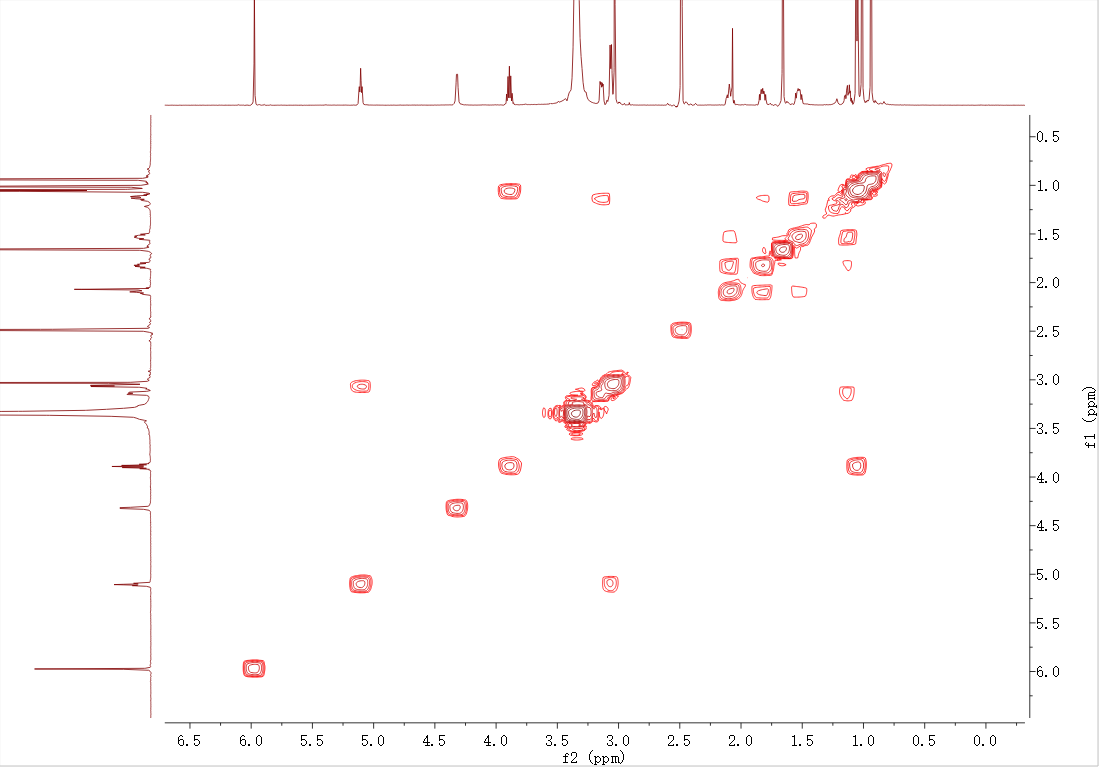


**Fig. S19** ^1^H–^1^H COSY spectrum of hyperfaberol C (**3)**.


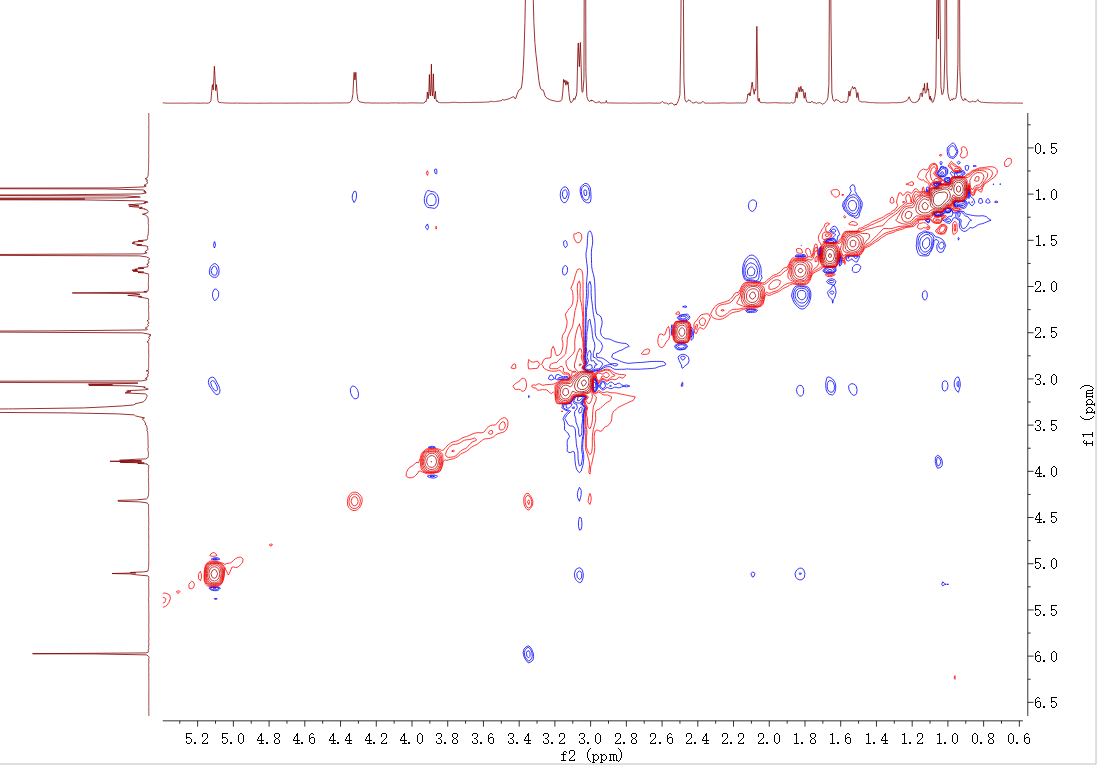


**Fig. S20** ROESY spectrum of hyperfaberol C (**3)**.


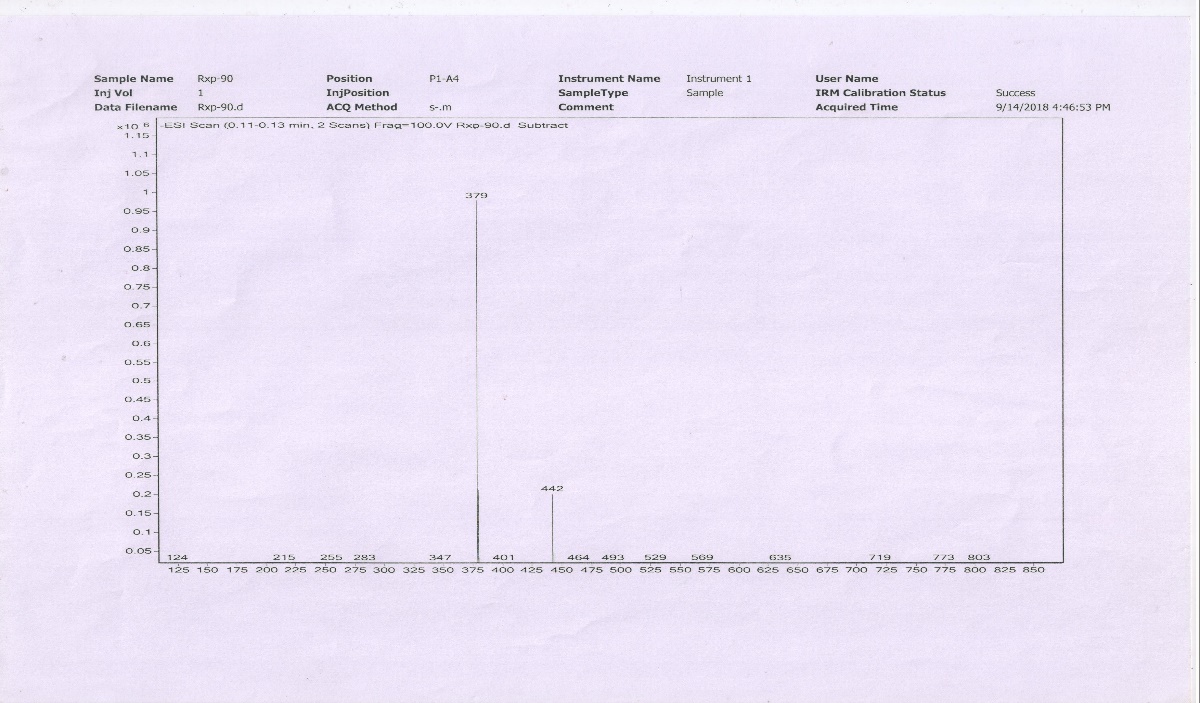


**Fig. S21** ESIMS spectrum of hyperfaberol C (**3)**.


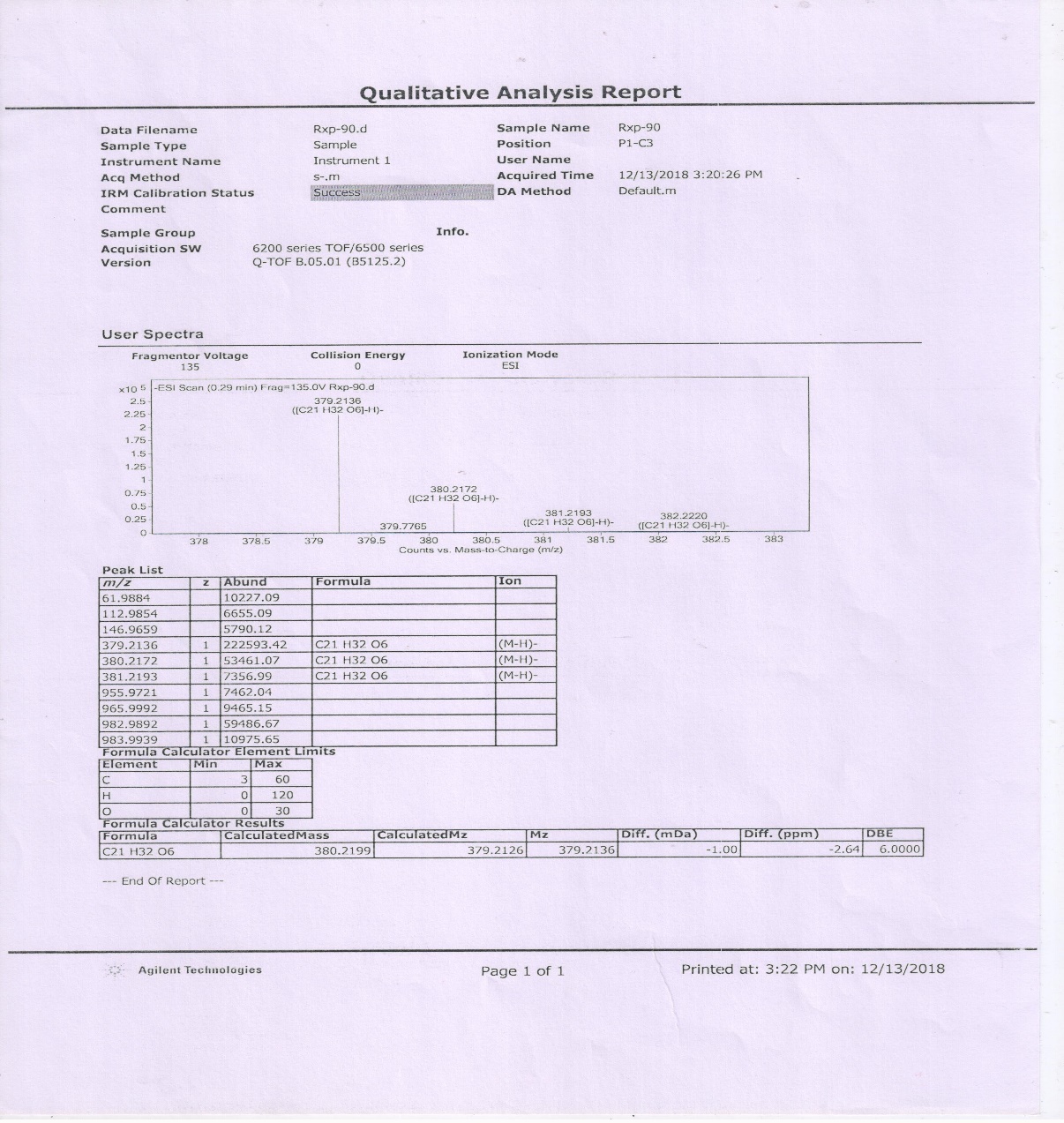


**Fig. S22** HRESIMS spectrum of hyperfaberol C (**3)**.


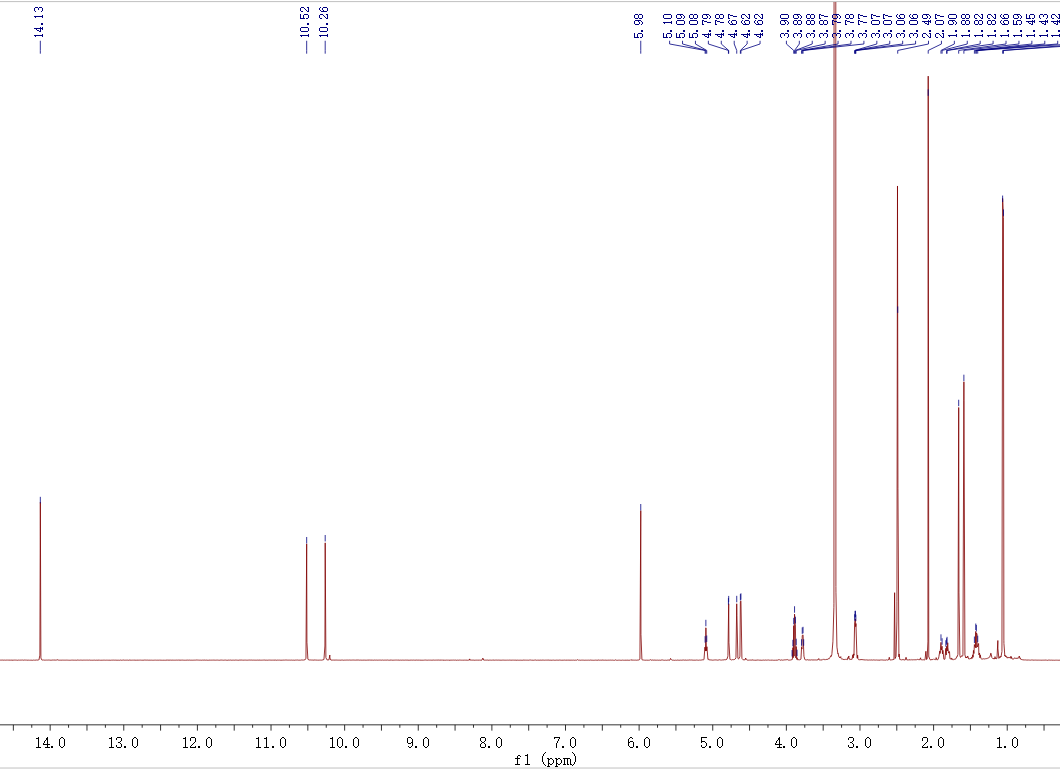


**Fig. S23** ^1^H (in DMSO-*d_6_*) spectrum of hyperfaberol D (**4)**.


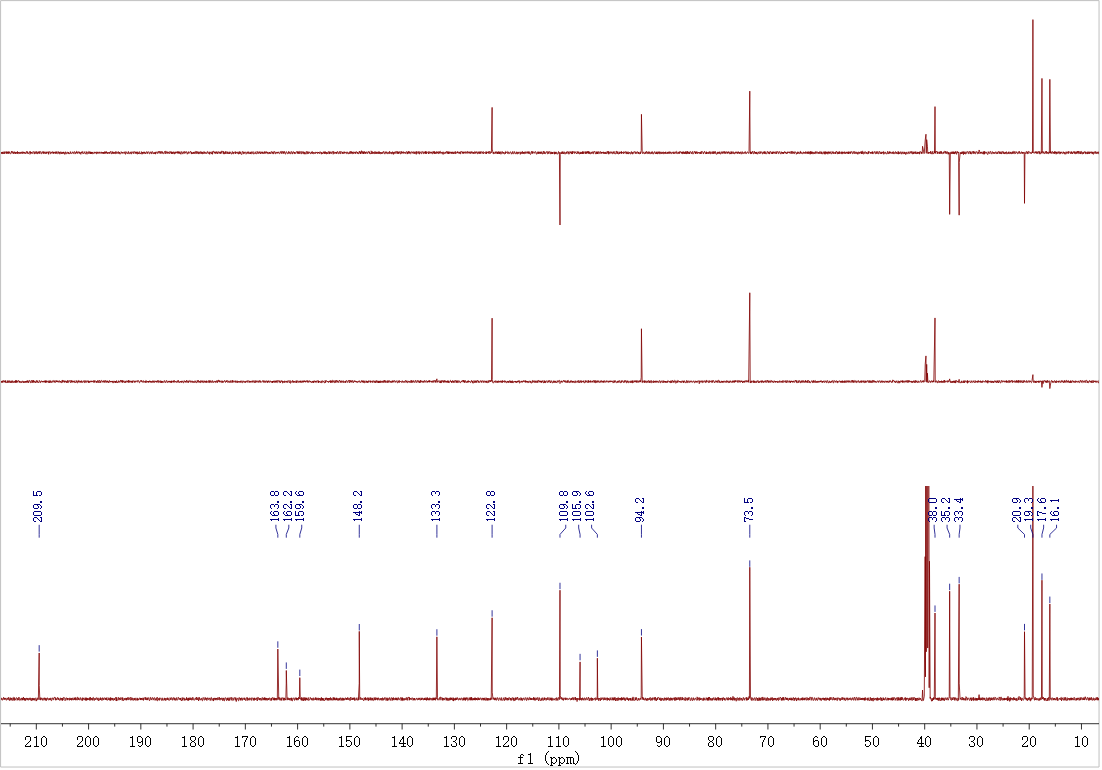


**Fig. S24** ^13^C and DEPT (in DMSO-*d_6_*) spectrum of hyperfaberol D (**4)**.


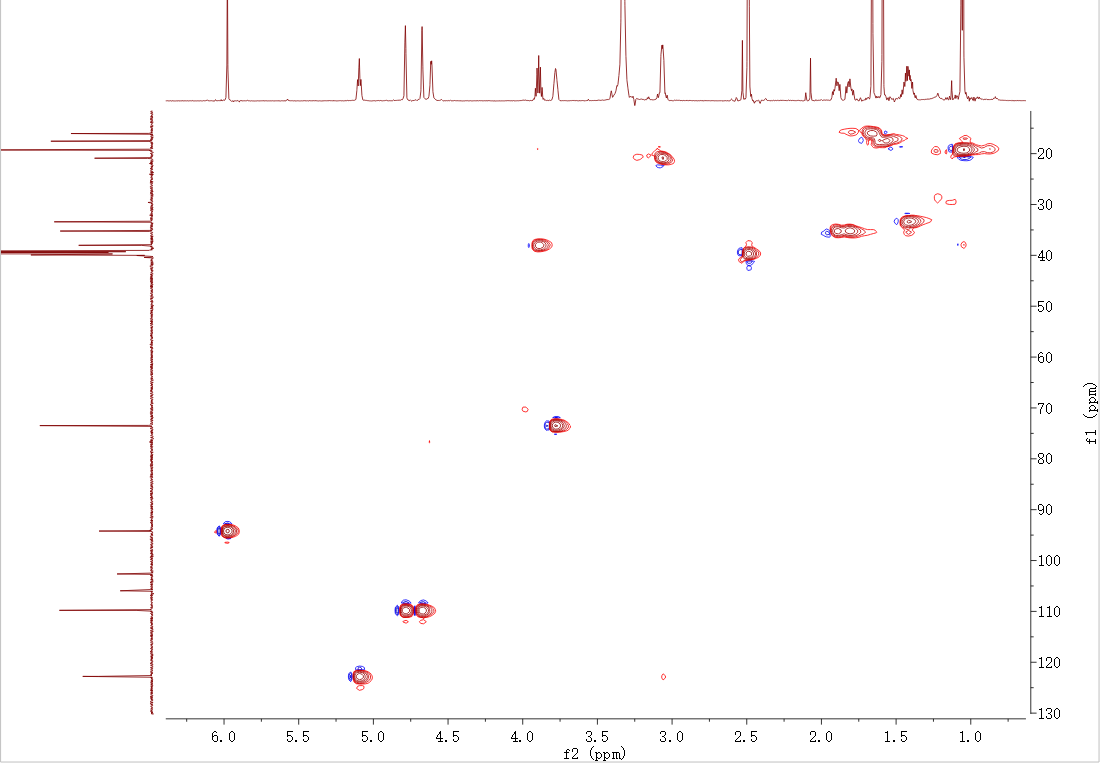


**Fig. S25** HSQC spectrum of hyperfaberol D (**4)**.


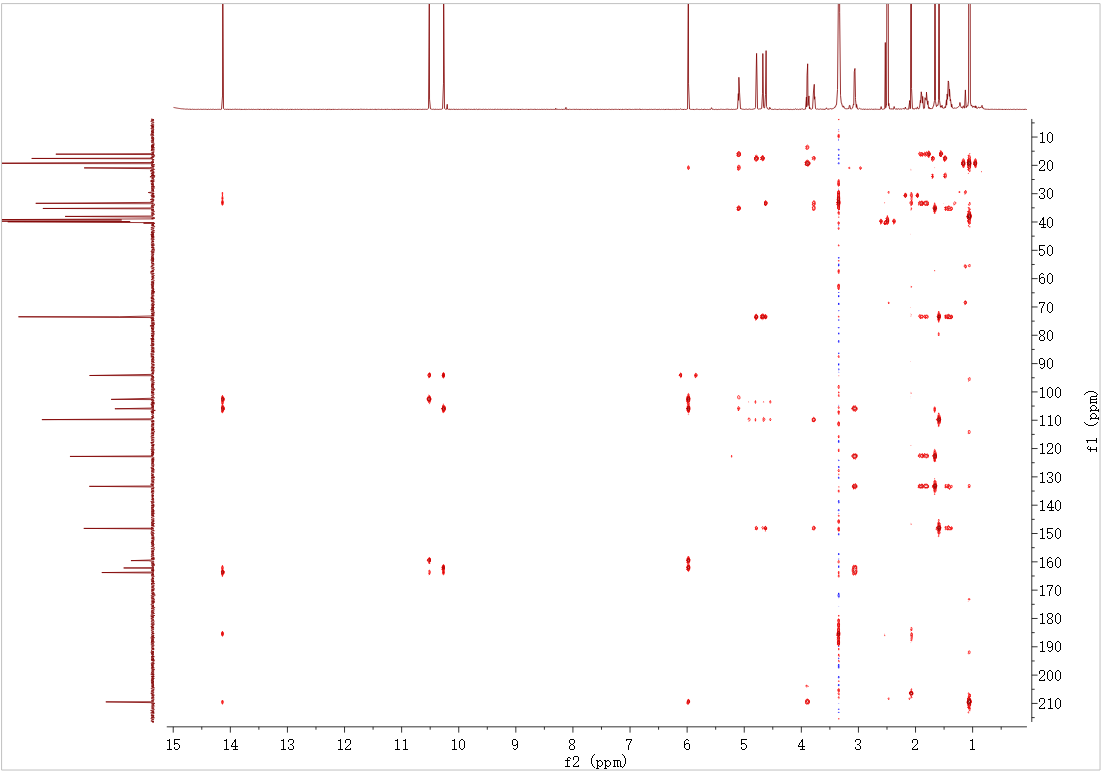


**Fig. S26** HMBC spectrum of hyperfaberol D (**4)**.


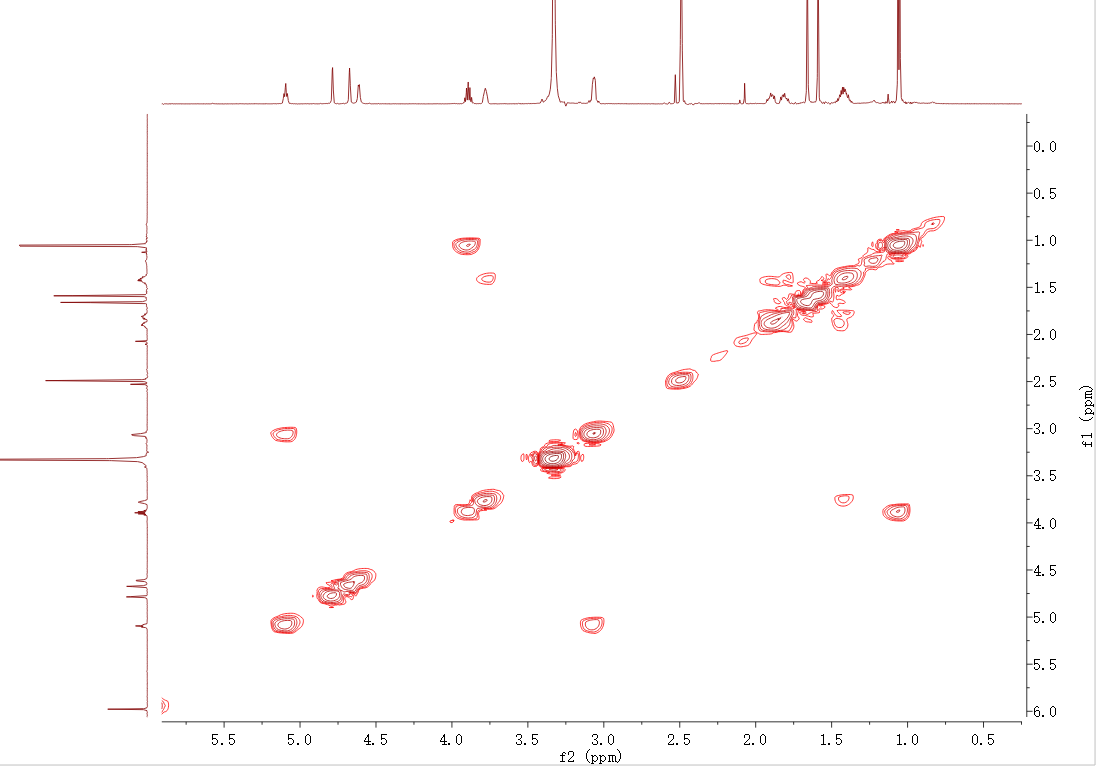


**Fig. S27** ^1^H–^1^H COSY spectrum of hyperfaberol D (**4)**.


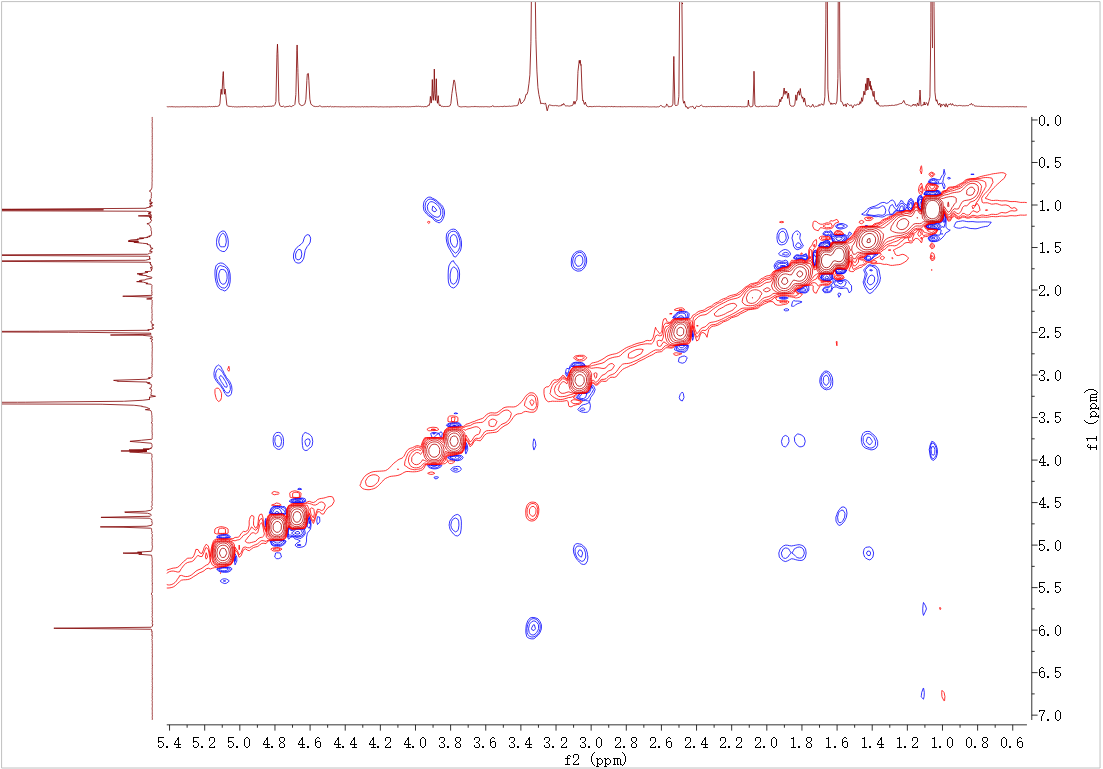


**Fig. S28** ROESY spectrum of hyperfaberol D (**4)**.


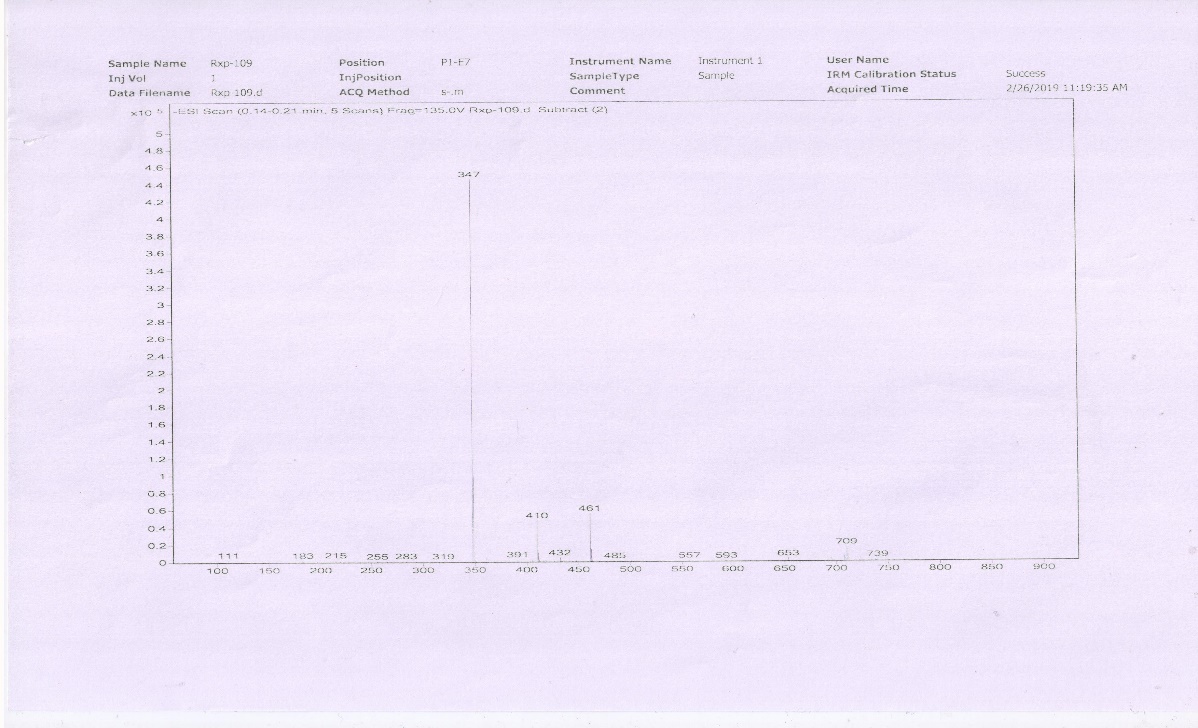


**Fig. S29** ESIMS spectrum of hyperfaberol D (**4)**.


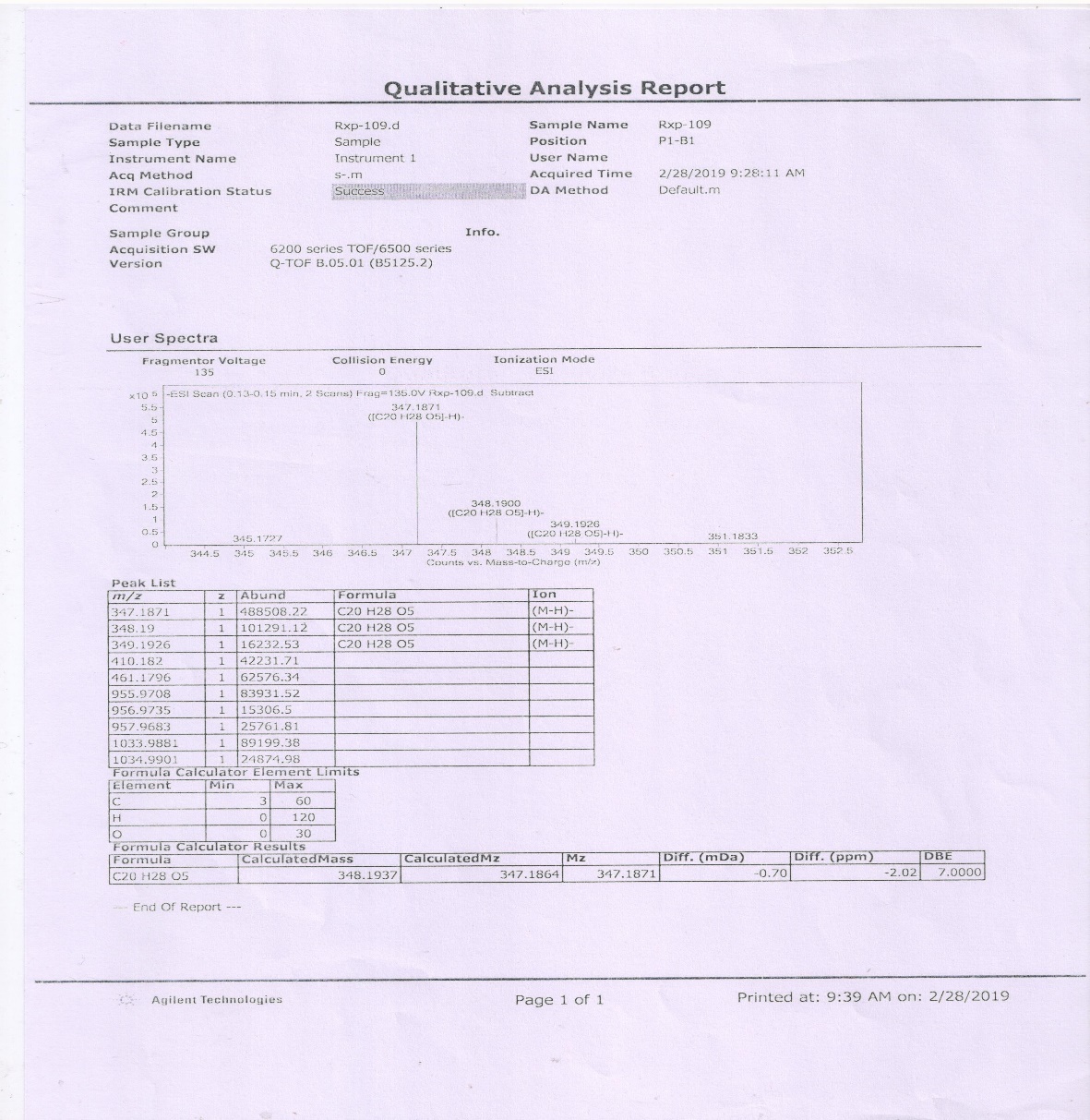


**Fig. S30** HRESIMS spectrum of hyperfaberol D (**4)**.


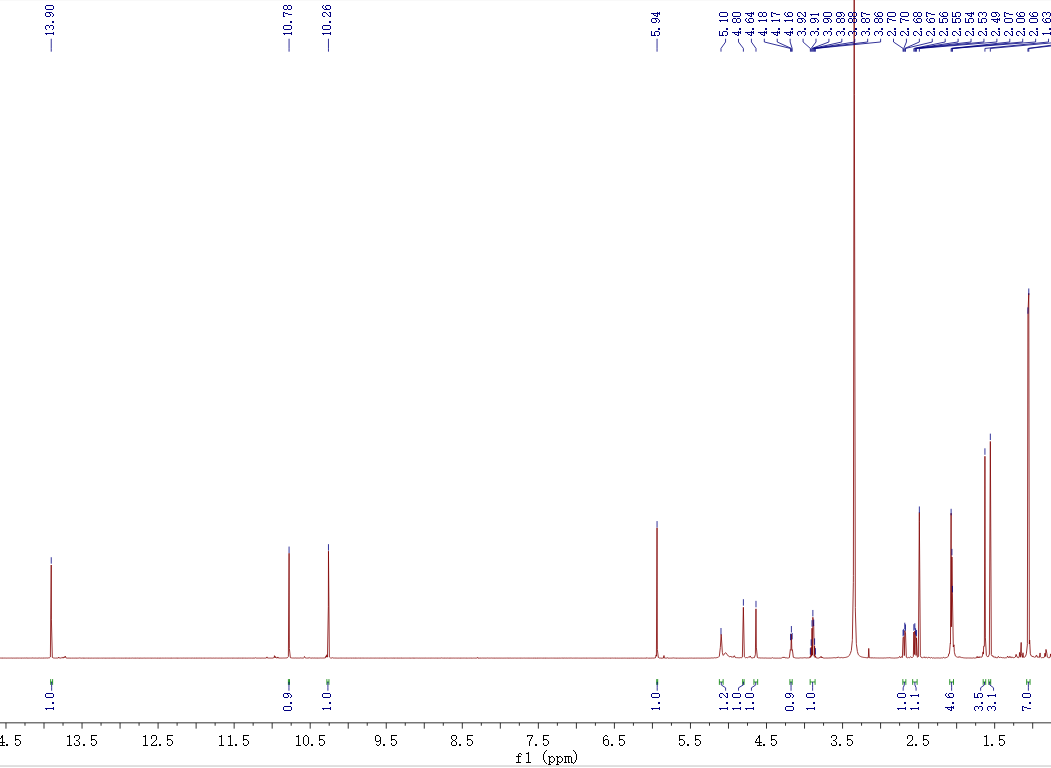


**Fig. S31** ^1^H (in DMSO-*d_6_*) spectrum of hyperfaberol E (**5)**.


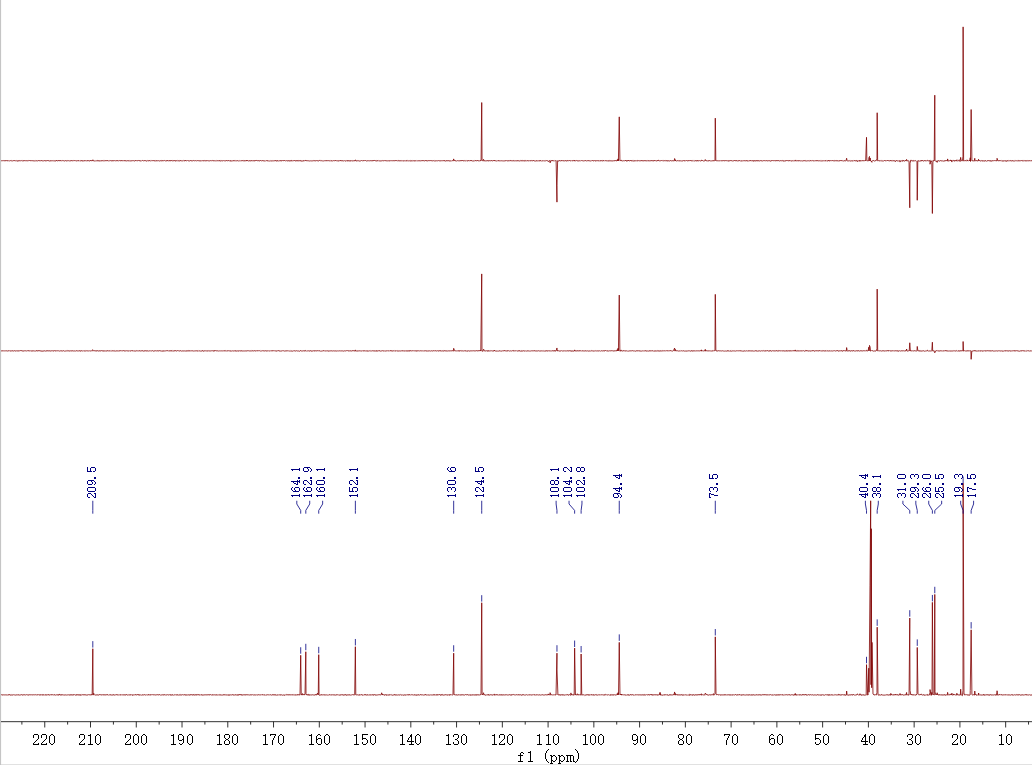


**Fig. S32** ^13^C and DEPT (in DMSO-*d_6_*) spectrum of hyperfaberol E (**5)**.


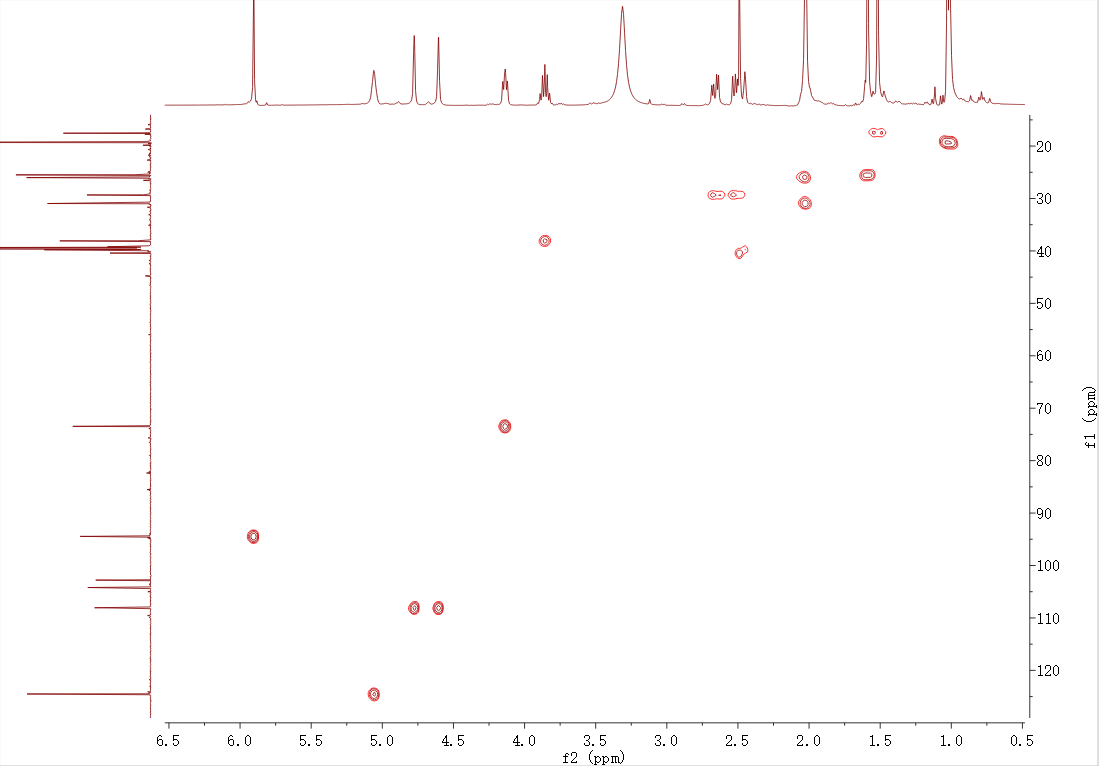


**Fig. S33** HSQC spectrum of hyperfaberol E (**5)**.


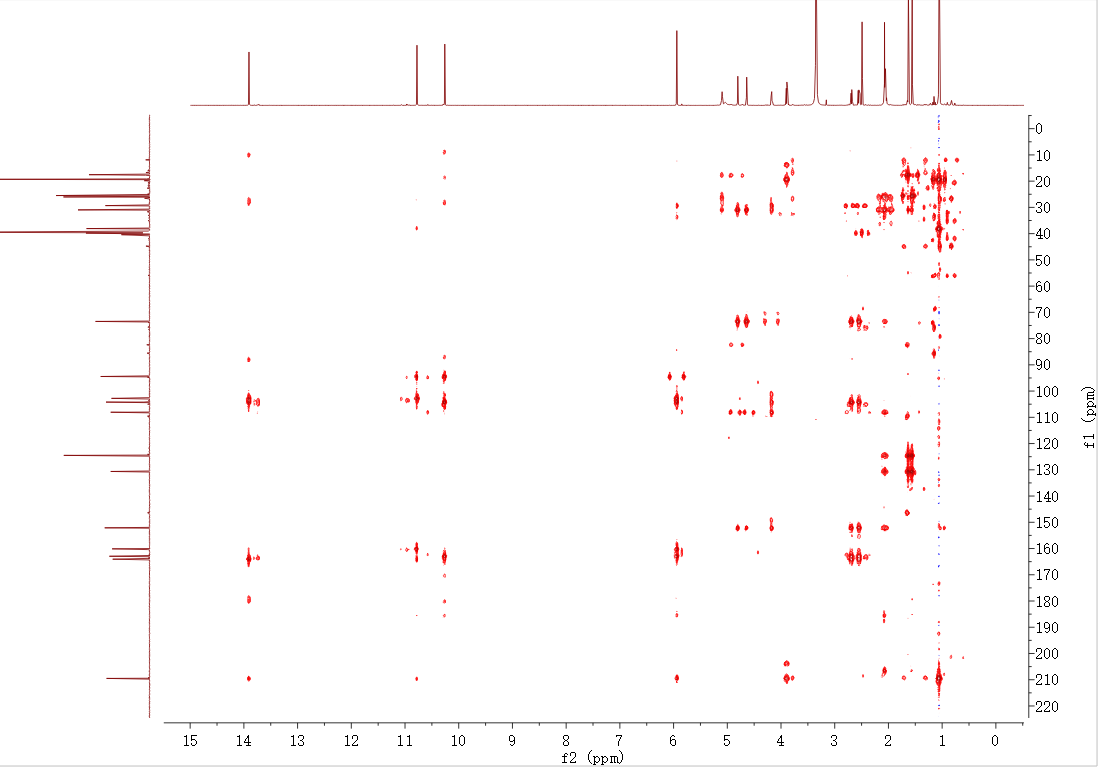


**Fig. S34** HMBC spectrum of hyperfaberol E (**5)**.


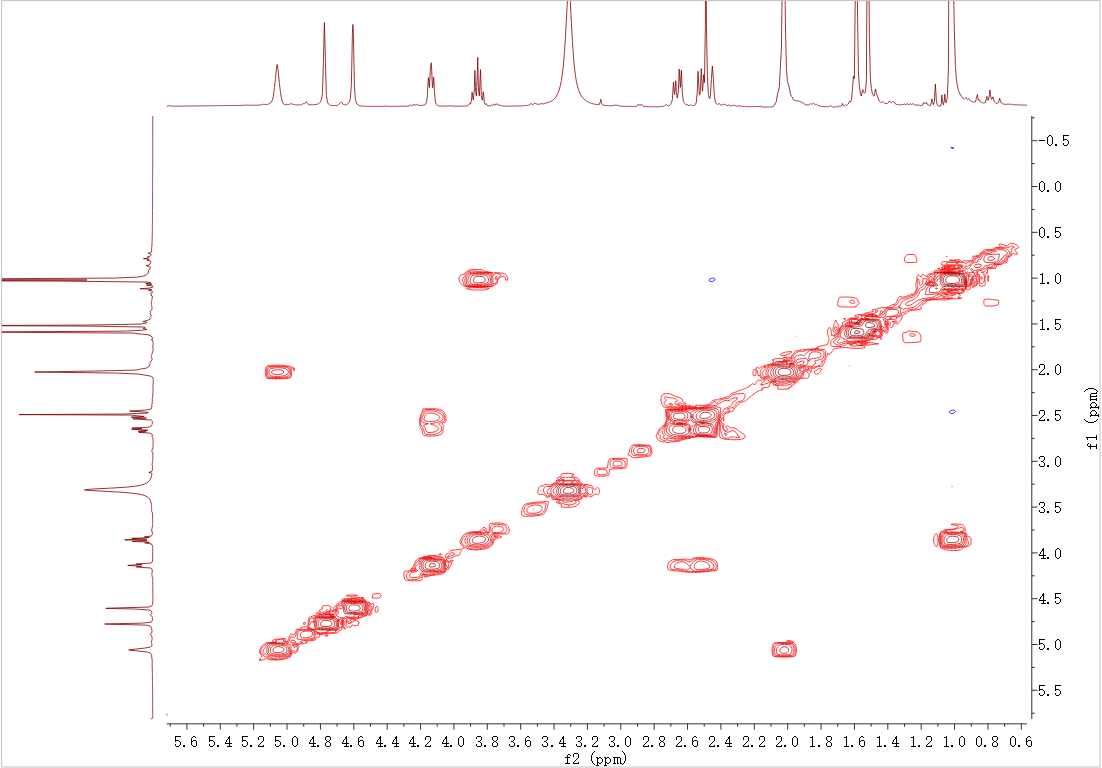


**Fig. S35** ^1^H–^1^H COSY spectrum of hyperfaberol E (**5)**.


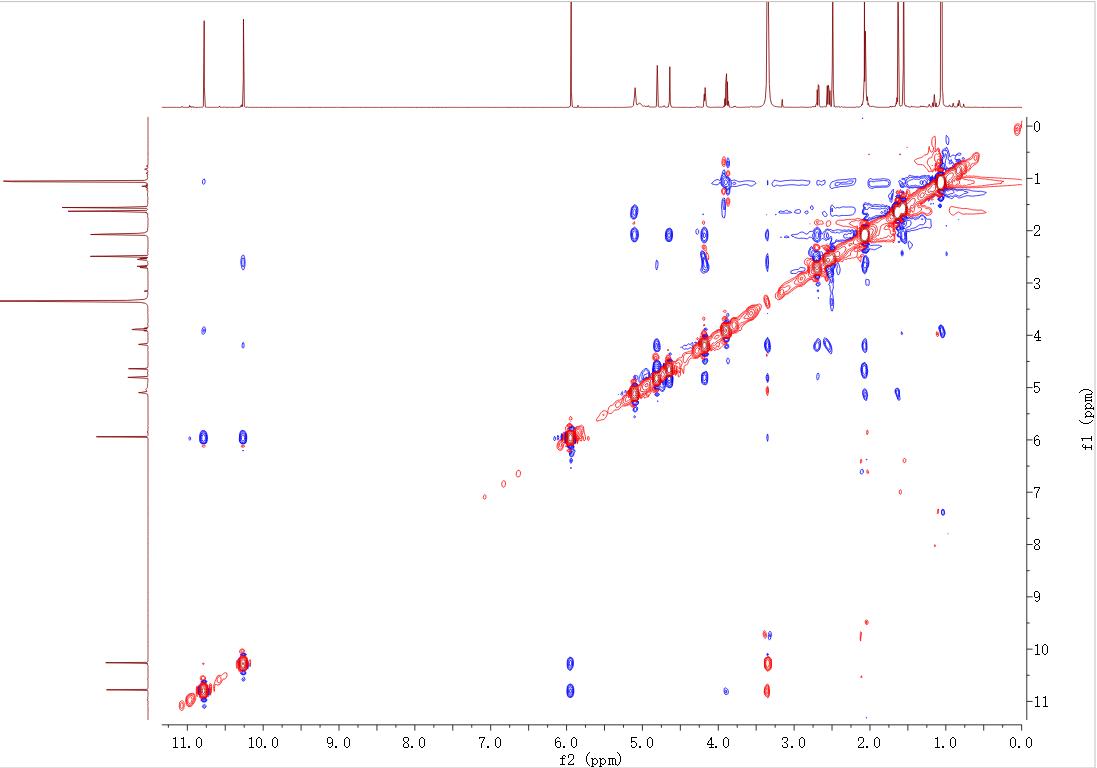


**Fig. S36** ROESY spectrum of hyperfaberol E (**5)**.


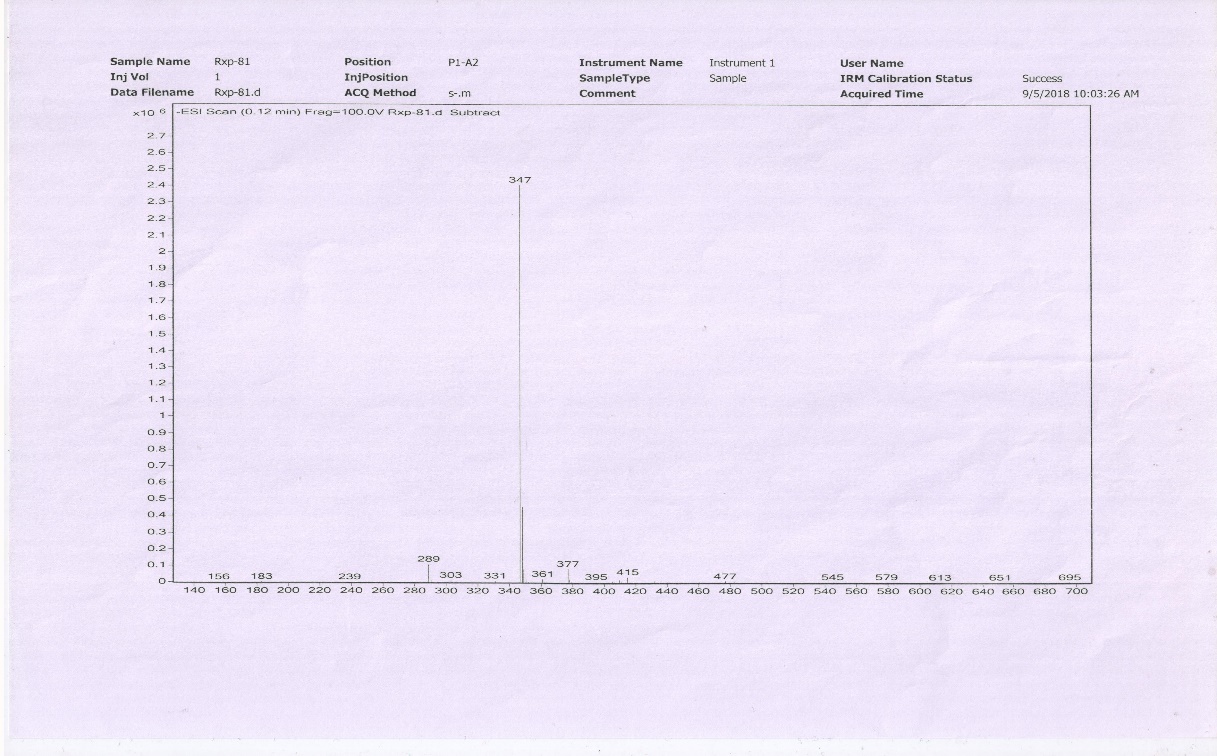


**Fig. S37** ESIMS spectrum of hyperfaberol E (**5)**.


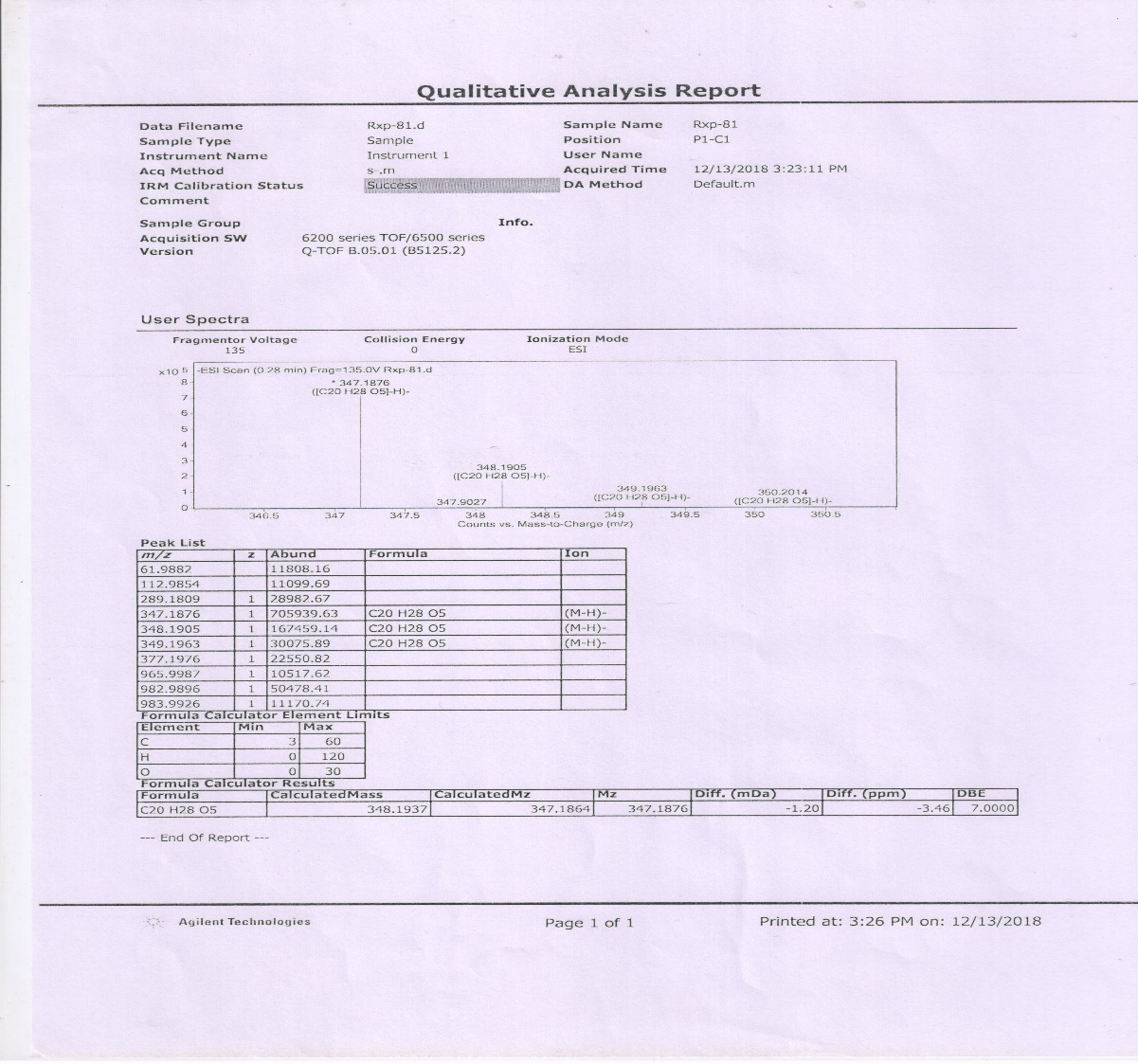


**Fig. S38** HRESIMS spectrum of hyperfaberol E (**5)**.


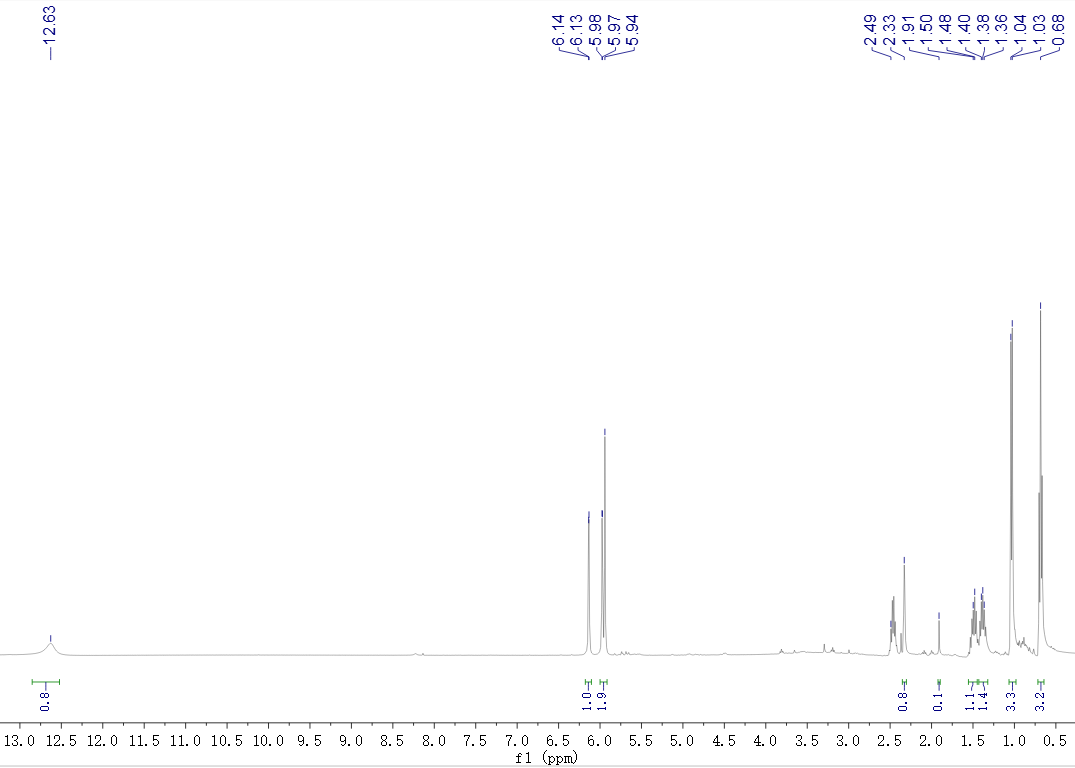


**Fig. S39** ^1^H (in DMSO-*d_6_*) spectrum of hyperfaberol F (**6)**.


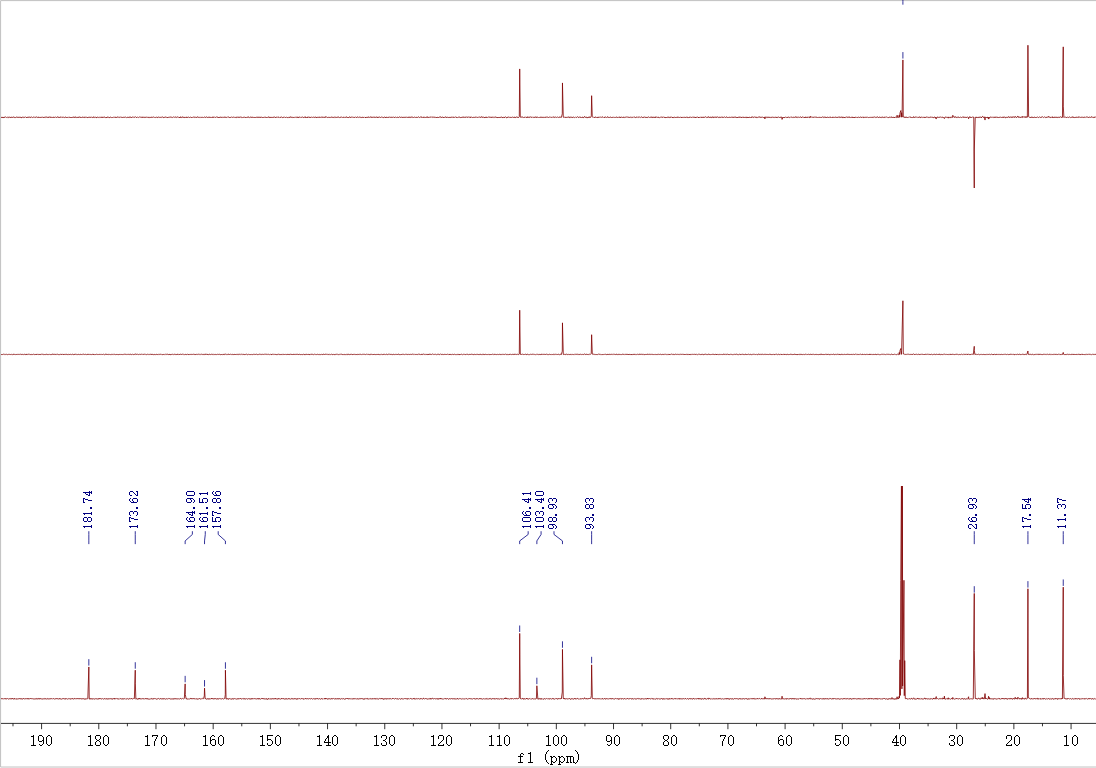


**Fig. S40** ^13^C and DEPT (in DMSO-*d_6_*) spectrum of hyperfaberol F (**6)**.


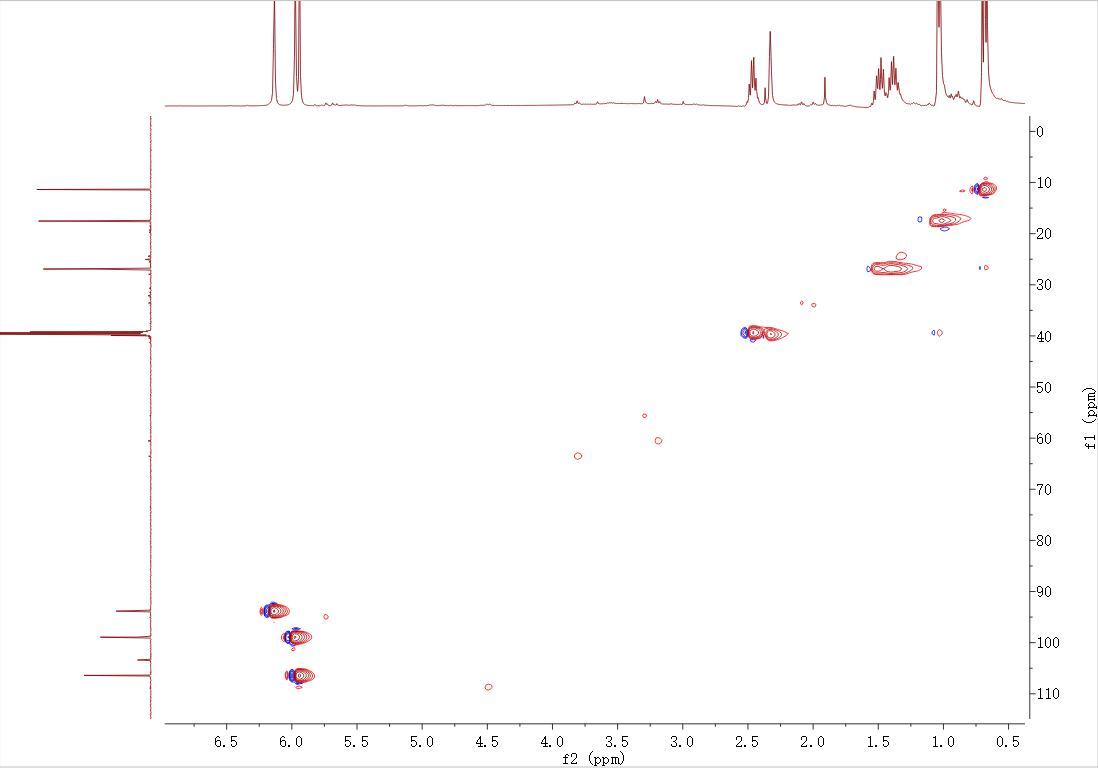


**Fig. S41** HSQC spectrum of hyperfaberol F (**6)**.


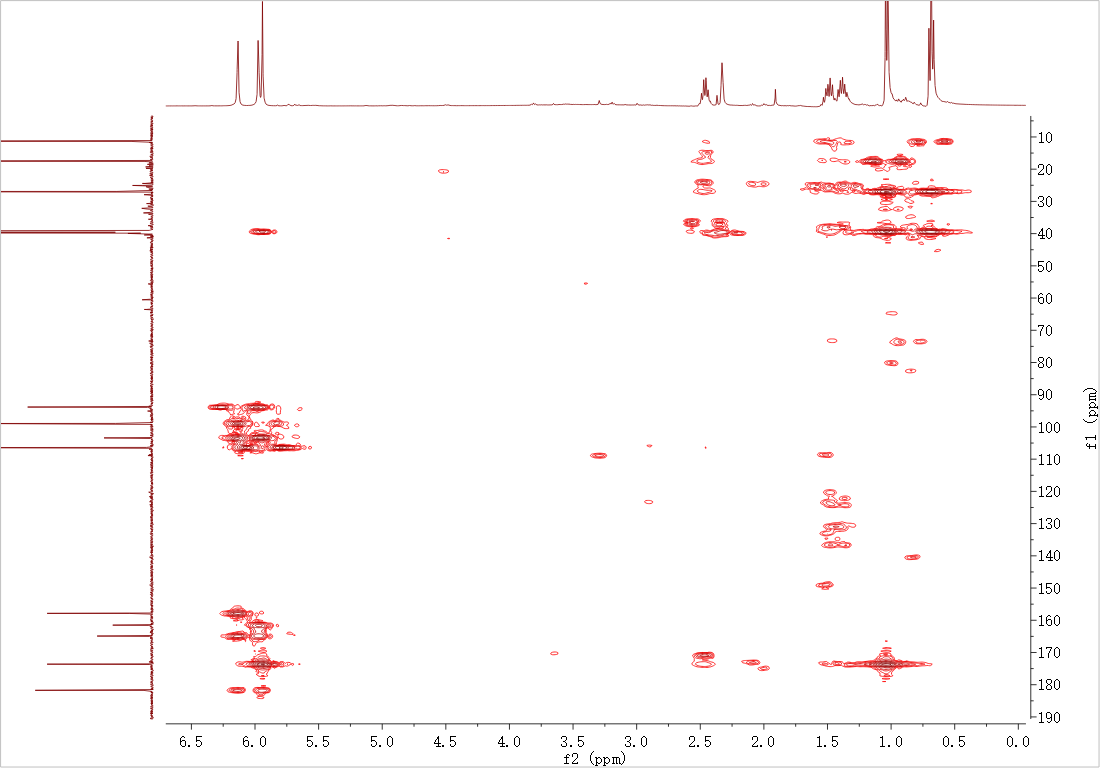


**Fig. S42** HMBC spectrum of hyperfaberol F (**6)**.


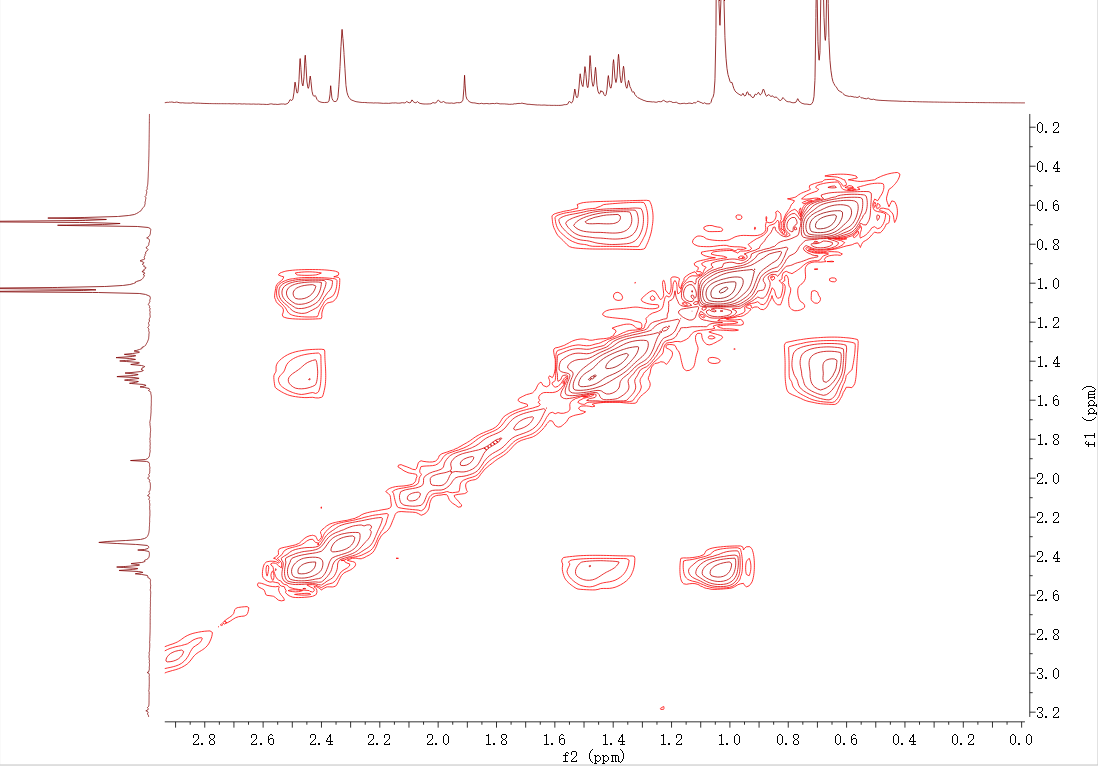


**Fig. S43** ^1^H–^1^H COSY spectrum of hyperfaberol F (**6)**.


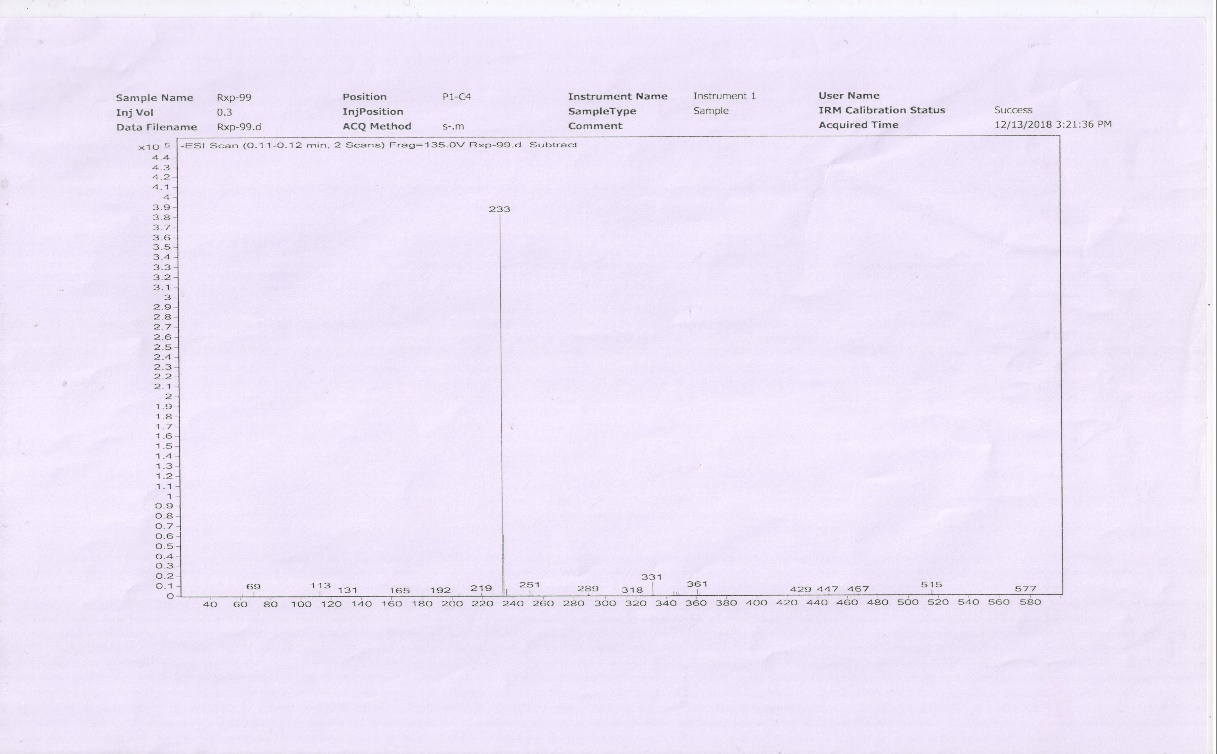


**Fig. S44** ESIMS spectrum of hyperfaberol F (**6)**.


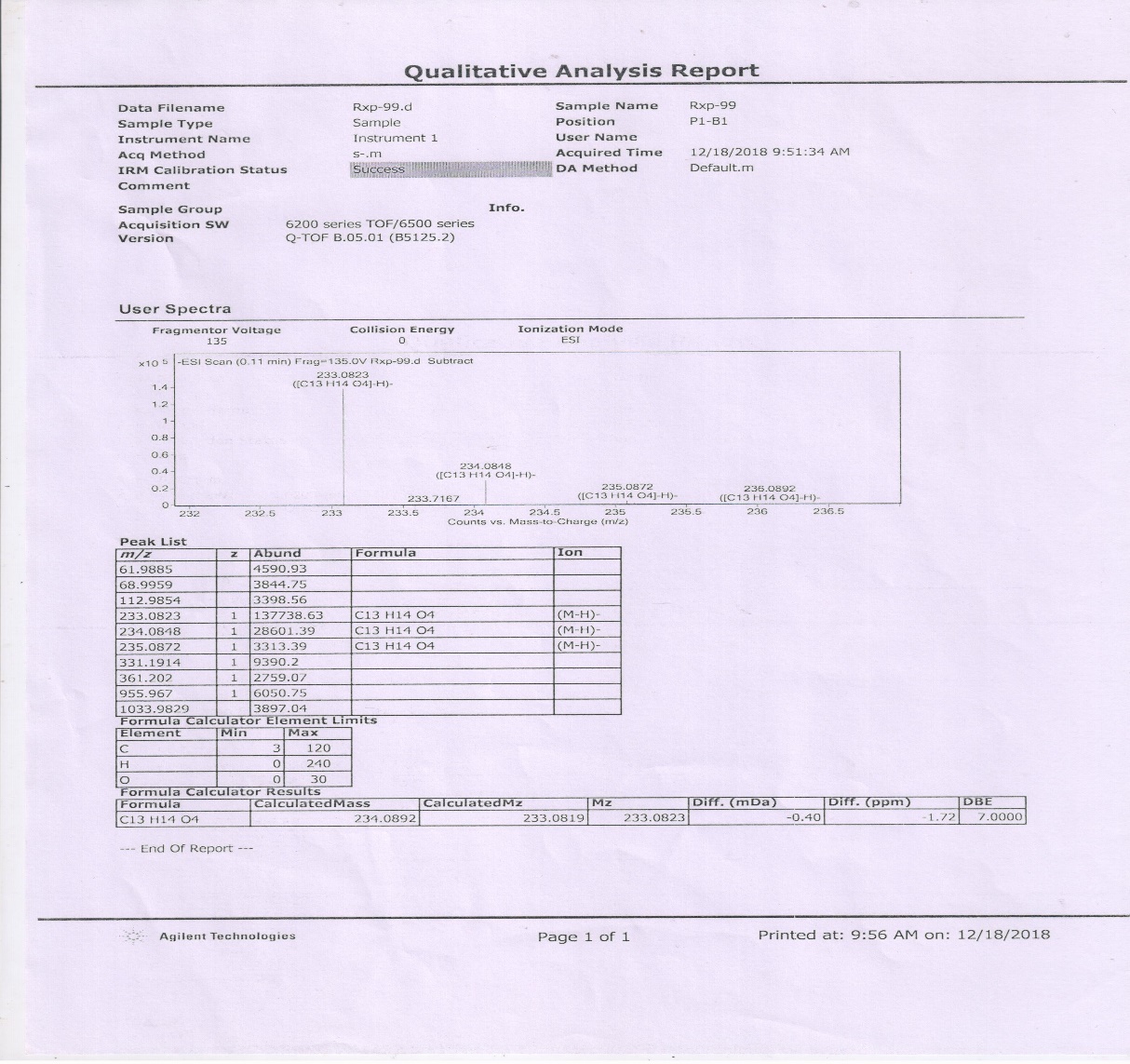


**Fig. S45** HRESIMS spectrum of hyperfaberol F (**6)**.
